# Molecular basis of rutin inhibition of protein disulfide isomerase (PDI) by combined *in silico* and experimental methods†

**DOI:** 10.1039/c8ra02683a

**Published:** 2018-05-21

**Authors:** Xu Wang, Guangpu Xue, Meiru Song, Peng Xu, Dan Chen, Cai Yuan, Lin Lin, Robert Flaumenhaft, Jinyu Li, Mingdong Huang

**Affiliations:** State Key Laboratory of Structural Chemistry, Fujian Institute of Research on the Structure of Matter, Chinese Academy of Sciences Fuzhou 350002 China HMD_lab@fzu.edu.cn +86 138 59021285; College of Life Science, Fujian Normal University Fuzhou 350117 China; College of Chemistry, Fuzhou University Fuzhou 350116 China j.li@fzu.edu.cn +86 188 60103237; College of Biological Science and Engineering, Fuzhou University Fuzhou 350116 China; Beth Israel Deaconess Medical Center, Harvard Medical School 330 Brookline Ave. Boston MA USA

## Abstract

Protein disulfide isomerase (PDI) is a founding member of the thiol isomerase family, and is recently found to play critical roles in thrombus formation. The development of effective PDI inhibitors is of great significance, and attracts strong interest. We previously showed that rutin bound directly to PDI and inhibited PDI activities, leading to the suppression of platelet aggregation and fibrin generation in a mouse model. A close analog of rutin, isoquercetin, is currently in advanced phase clinical trials. However, the molecular interaction between rutin and PDI is unknown and is difficult to study by X-ray crystallography due to the weak interaction. Here, we generated a molecular model of PDI:rutin complex by molecular docking and thorough molecular dynamics (MD) simulations. We then validated the complex model through a number of different experimental methods. We mutated the key residues predicted by the model and analyzed the mutants by an optimized isothermal titration calorimetry (ITC) method and a functional assay (insulin reduction assay). The results consistently showed that the PDI residues H354, L355 and E359 are important in the binding of rutin. These residues are next to the canonical major substrate binding site of the b′ domain, and were not conserved across the members of thiol isomerases, explaining the specificity of rutin for PDI among vascular thiol isomerases. Furthermore, the inhibitory activities of three rutin analogues were evaluated using an insulin reduction assay. The results supported that the second sugar ring at the side chain of rutin was not necessary for the binding to PDI. Together, this work provides the structural basis for the inhibitory mechanism of rutin to PDI, and offers a promising strategy for the design of new generation inhibitors with higher binding affinity to PDI for therapeutic applications.

## Abstract 2

Rutin binds and inhibits PDI at b′x domain, H354 is one of the main binding sites.

## Graphical abstract

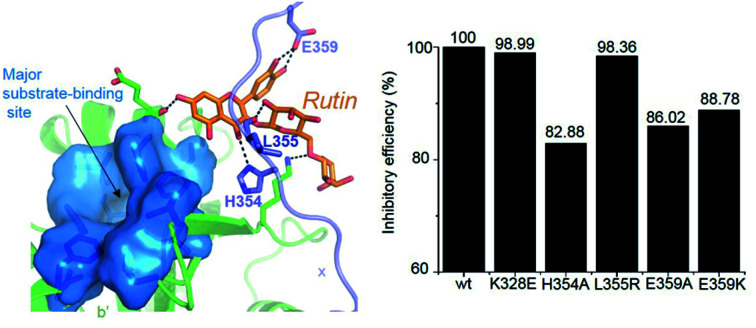

## Introduction

Protein disulfide isomerase (PDI; EC 5.3.4.1) is a member of the thioredoxin superfamily of redox proteins,^1^ which contains more than 20 different members.^2^ PDI normally exists and functions in endoplasmic reticulum (ER),^3^ but under certain circumstances can escape from ER to other locations, *e.g.* plasma membrane.^4^ PDI can be either an oxidoreductase or a molecular chaperone.^5^ PDI facilitates the folding of nascent proteins or misfolded proteins to ensure proper disulfide bond formation through its oxidase and reductase activities.^6^

**Fig. 1:**
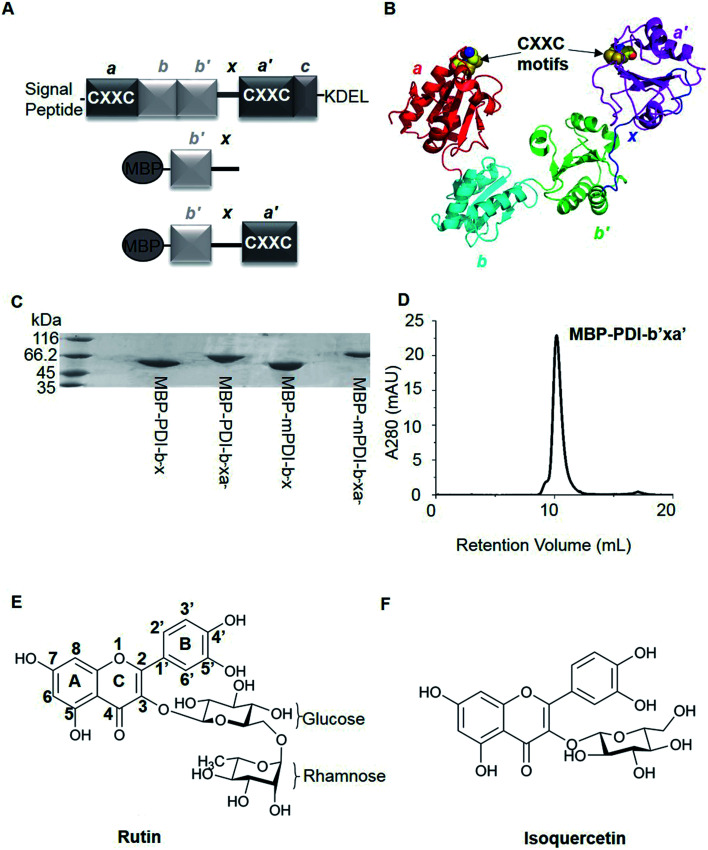
Overviews of PDI proteins and small-molecule inhibitors mentioned in this paper. (A) Domain organization diagram of PDI and MBP-fused PDI proteins. PDI contains four thioredoxin-like domains, labeled as a, a′, b and b′ from the amino terminal to the carboxyl terminal. There are additional x linker between b′ and a′ domains and short C-terminal extension after a′ domain. In addition, a and a′ domains have a conserved sequence, termed CXXC motif. And normally, there are signal peptide sequence at the N-terminus and KDEL at the C-terminus of PDI. For MBP-fused PDI, MBP was fused at the N-terminus of PDI. We constructed b′x and b′xa′ recombinant proteins of human and murine PDI, namely MBP-PDI-b′x, MBP-PDI-b′xa′, MBP-mPDI-b′x and MBP-mPDI-b′xa′, respectively. MBP-fused PDI proteins were isolated by affinity chromatography with the His-tag purification resin and further purified by gel filtration chromatography. (B) Cartoon representation of the crystal structure of human PDI (abb′xa′, PDB ID: 4EL1). The a, b, b′ and a′ domains were colored red, cyan, green and magenta, respectively. The x linker was displayed in blue. The four cysteine residues of the two active sites (CXXC motifs) were represented by spheres. (C) The reduced SDS-PAGE analysis of the purified MBP-fused PDI proteins. The molecular weight of MBP-PDI-b′x, MBP-PDI-b′xa′, MBP-mPDI-b′x and MBP-mPDI-b′xa′ are 59.4 kDa, 71.8 kDa, 59.2 kDa and 71.4 kDa, respectively. (D) Analysis of the purified wt MBP-PDI-b′xa′ by gel filtration chromatography on Superdex75. (E) and (F), chemical structure of rutin (E) and isoquercetin (F). Rutin and isoquercetin has typical structure of flavonoid with two benzene rings (A and B rings) and a pyranic ring (C ring). Isoquercetin is glycosylated at position 3, while rutin core is attached with a rhamnose and a glucose at position 3.

PDI is composed by multiple domains, including a short signal peptide at the amino terminal, four thioredoxin-like domains (namely a, b, b′, and a′), one linker between b′ and a′ domains (x linker), a short acidic C-terminal extension and the ER retention signal sequence (KDEL) at the carboxyl terminal (Fig. 1A).^7^ The a and a′ domains of PDI are considered to be the catalytic domains containing the conserved active CXXC motifs.^8^ The b and b′ domains of PDI are thought to function as substrate recognition and binding sites.^9^

PDI is involved in a number of physiological and pathological processes.^10^ It has been demonstrated that PDI is overexpressed in diseases with ER stress.^11,12^ PDI is a promising target for cancer therapy.^13^ PDI has also been shown to play a vital role in thrombus formation.^14^ PDI is rapidly secreted from platelets and endothelial cells and is critical for platelet accumulation and fibrin generation at sites of injury in a mouse thrombosis model, demonstrating that PDI is also a feasible target for antithrombotic treatment.^15^

There has been great interest in developing PDI inhibitors in the past few decades. A few types of PDI inhibitors were reported previously, which include antibodies, peptides, and small organic molecules.^16–19^ Many of the small molecular inhibitors covalently bind to the catalytic cysteine residues within the active sites and are irreversible inhibitors of PDI. It is also a challenge to develop specific small-molecule inhibitors of PDI as the majority of the inhibitors act broadly on other thiol isomerases. Rutin (Fig. 1E) was found to be a specific and reversible inhibitor of PDI.^20^ Rutin selectively bound and inhibited PDI activities in a dose-dependent way with an IC_50_ of at micromolar range.^20,21^ Although the interaction between rutin and PDI is weaker than other small-molecule inhibitors, for example, juniferdin that inhibits PDI at 156 nM,^22^ the *in vivo* inhibitory effect of rutin on PDI was well documented in a number of studies. Inhibition of PDI by rutin blocked both platelet aggregation and fibrin generation *in vivo* at a dose of 0.5 mg kg^−1^ in mouse models. Furthermore, the inhibitory activities of rutin on PDI can be rapidly reversed *in vivo* by the infusion of PDI b′x fragment in mouse model.^21^ Recently, isoquercetin, a close analogue of rutin with only one glucose moiety in its structure (Fig. 1F), was tested on human through oral administration (1 gram) and was demonstrated to reduce plasma thrombin generation activity by about half through inhibition of PDI.^23^

Even though rutin appears to be a potential antithrombotic agent, it still has low potency and low bioavailability. In addition, the detailed molecular interaction between PDI and rutin is still largely unknown, and is difficult to study by X-ray crystallography, partly due to the weak nature of this interaction. We previously localized the binding site of rutin to the b′xa′ fragment of PDI.^21^ In this study, we used a combined molecular simulation and molecular biology method to tackle this problem by first generating a molecular model of rutin binding to PDI through thorough computational study, and validating the model using rigorous experimental assays. The findings obtained demonstrated the feasibility of this approach, and provided a structural template to design better and more potent inhibitors of PDI.

## Materials and methods

### 1. Materials

Mouse P4HB Gene ORF cDNA clone in cloning vector purchased from SinoBiological Inc. Constructed plasmid vector pMCSG9 (containing His-tag, TEV site and MBP) and *Escherichia coli* (*E. coli*) BL21 (DE3) cells were from our laboratory. Rutin was purchased from Sangon Biotech (Shanghai) Co., Ltd. Kaempferitrin was purchased from Chengdu Micxy Chemical Co., Ltd. Tiliroside was purchased from Shanghai Yuanye Bio-Technology Co., Ltd. 2′-*O*-Galloylhyperin was purchased from Chengdu Herbpurify Co., Ltd. Insulin was purchased from Shanghai Yeasen Bio-Technology Co., Ltd. We analysed the purity of these small-molecule chemicals by HPLC (Fig. S1†). All these chemical reagents have high purity for scientific study.

### 2. Ligation independent clone (LIC)

Human PDI (hPDI) and murine PDI (mPDI) genes were cloned into a pMCSG9 plasmid vector through ligation independent clone (LIC). The human MBP-PDI-b′x (235–369) and MBP-PDI-b′xa′(235–479) was generated using a forward primer: 5′ TACTTCCAATCCAATGCTCCCCTTGTCATCGAGTTCAC 3′ and reverse primers: 5′ TTATCCACTTCCAATGTTAGACAGGCTGCTTGTCCCAGT 3′ for b′x and 5′ TTATCCACTTCCAATGTTAATCCCCTGCCCCATCC 3′ for b′xa′. The murine MBP-mPDI-b′x (236–369) and MBP-mPDI-b′xa′ (236–477) was generated using a forward primer: 5′ TACTTCCAATCCAATGCTCTGCCTTTGGTCATCGAGTTCAC 3′ and reverse primers: 5′ TTATCCACTTCCAATGTTACTGTTTGTCCCAGTCTT 3′ for b′x and 5′ TTATCCACTTCCAATGTTACTGGCCACCACTCTCCAAGAATTTC 3′ for b′xa′. The pMCSG9 was linearized by *SspI* (Takara Inc.), then the PCR products and the pMCSG9 were simultaneously treated with T4 DNA polymerase together with dGTP/dCTP. The annealed products were transformed into *E. coli* BL21 (DE3) cells. Synthesis of the primers and DNA sequencing were carried out by Biosune Co., Ltd.

### 3. Site-directed mutagenesis

Site-directed mutant proteins were cloned where the PCR template was PDI b′xa′ domain containing MBP (MBP-PDI-b′xa′) on pMCSG9 plasmid vector. We used full-length pMCSG9 plasmid as template to carry out site-directed mutagenesis rapidly. Primers: (A) K328E: the forward primer, 5′ CCAAGTACGAGCCCGAATCGGAGGAG 3′, and the reverse primer, 5′ TTCGGGCTCGTACTTGGTCATCTCCT 3′; (B) H354A: the forward primer, 5′ CAAGCCCGCCCTGATGAGCCAGGAG 3′, and the reverse primer, 5′ CATCAGGGCGGGCTTGATTTTGCC 3′; (C) L355R: the forward primer, 5′ AAGCCCCACAGGATGAGCCAGGAGCTG 3′, and the reverse primer, 5′ GCTCATCCTGTGGGGCTTGATTTTGCC 3′; (D) E359A: 5′ GAGCCAGGCGCTGCCGGAGGACTGGG 3′, and the reverse primer, 5′ CCGGCAGCGCCTGGCTCATCAGGTGG 3′; (E) E359K: 5′ TGAGCCAGAAGCTGCCGGAGGACTGG 3′, and the reverse primer, 5′ CGGCAGCTTCTGGCTCATCAGGTGGG 3′. Primers where the mutant sites are at the center were amplified the full-length pMCSG9 plasmid using Phusion High-Fidelity DNA polymerase (ThermoFisher Scientific Inc.) or Q5 High-Fidelity DNA polymerase (New England BioLabs Inc.). Then the non-mutant pMCSG9 plasmids were digested by *SspI* (Takara Inc.) while mutants were transformed into *E. coli* BL21 (DE3) cells.

### 4. Expression and purification of recombinant proteins

MBP fusion proteins expressed in *E. coli* strain BL21 (DE3) after they were induced by isopropyl-β-d-thiogalactoside (IPTG). All recombinant proteins were captured by affinity chromatography with His-tag purification resin (Chelating Sepharose Fast Flow from GE Healthcare), followed by gel filtration chromatography through Superdex75 HR 10/300 column (GE Healthcare) equilibrated with protein buffer (pH 7.4) (20 mM Tris containing 150 mM NaCl, 1 mM ethylenediaminetetraacetic acid (EDTA), 1 mM β-mercaptoethanol (β-ME) and 5% glycerin). The purified recombinant proteins were stored at −80 °C.

### 5. Fluorescence-based direct binding assay

The relevant recombinant proteins were incubated with rutin in black 384-well plate at room temperature for 30 min before measurements. The concentrations of proteins were always kept at 18 μM and rutin was 55 μM. The total assay volume was filled to 100 μL using the protein buffer (pH 7.4). The fluorescence emission spectrum of every mixture were measured with the excitation at 430 nm and the emission at 460 nm at room temperature on a BioTek Synergy microplate reader with a sensitivity of 100 or 120 and measurements of per data with 100 times.

### 6. Insulin reduction assay

The insulin reduction assay was performed to assess the enzymatic activity of PDI and its MBP-fused recombinant proteins. PDI can reduce insulin in the presence of dithiothreitol (DTT) along with increase in turbidity of the reaction mixture. We thus monitored spectrophotometrically the aggregation of reduced insulin through the increase of turbidity. The reductase activity was measured in 100 μL in presence of 600 μM insulin, 1.5 μM PDI and recombinant PDI proteins, 1 mM DTT, and with protein buffer (pH 7.4), at room temperature over 100 min by absorption at 650 nm. For inhibition assay, 100 μM of rutin, kaempferitrin, tiliroside and 2′-*O*-galloylhyperin or control buffer was added to the reaction system.

### 7. Isothermal titration calorimetry (ITC)

Microcalorimetry titration of rutin to PDI recombinant and mutant proteins used a MicroCal ITC200 microcalorimeter (GE Healthcare). PDI recombinant proteins, which concentration ranges from 25 μM to 50 μM, were added into the cell (about 300 μL). Rutin, with concentration ranging from 500 μM to 1 mM, was injected from syringe to titrate the protein in cell (almost 40 μL). The experiment was performed at 25 °C with a equilibration time of 10 min, the reference power was set to 6.25 μcal s^−1^. The process including 25 injections, each of 1, 2 or 2.5 μL rutin, in a duration of 3 s, and the interval time between two injections was 180 s. Data was analyzed with MicroCal Origin (MicroCal).

### 8. Molecular docking

The molecular docking calculations of rutin onto PDI were performed with AutoDock 4.2 package^24^ using the Lamarckian genetic algorithm.^25^ The initial 3D structures of PDI was obtained from the PDB database (PDB ID: 4EL1,^26^ resolution at 2.88 Å). The geometry of rutin was optimized using GAMESS^27^ with the B3LYP functional^28^ and 6-31G(*) basis set.^29^ The grid map, centered at PDI was calculated using 126 × 126 × 126 points with 0.375 Å spacing. Two-thousand docking runs were carried out. The binding energy and cluster analyses were conducted using the default parameters implemented in AutoDockTools 1.5.^24^ The best docking result was defined by the model that has the best binding affinity within the dominant cluster.

### 9. Molecular dynamics (MD) simulation

The model of PDI in complex with rutin obtained from docking calculations was inserted into box of explicit water and 150 mM NaCl (corresponding to the salt concentration used in our binding assays). The protonation states of residues were assigned according to the corresponding p*K*_a_ values calculated by using the H++ webserver.^30^ Sodium ions were added to counterbalance the charge of the complex. The system contained 86 132 atoms in total. It was underwent MD simulations using the GROMACS 4.6.5 code^31^ on in-house GPU clusters. The force fields of PDI and rutin were set to AMBER ff99SB-ILDN force field^32–34^ and general AMBER force field (GAFF),^35^ respectively. The Åqvist potential^36^ and TIP3P model^37^ were used for the ions and for the water molecules, respectively. All bond lengths were constrained by LINCS algorithm.^38^ Periodic boundary conditions were applied. Electrostatic interactions were calculated using the Particle Mesh-Ewald (PME) method,^39^ and van der Waals and Coulomb interactions were truncated at 10 Å. The system underwent 1000 steps of steepest-descent energy minimization with 1000 kJ mol^−1^ Å^−2^ harmonic position restraints on the protein, followed by 2500 steps of steepest-descent and 2500 steps of conjugate-gradient minimization without restraints. The system was then gradually heated from 0 K up to 298 K in 20 steps of 2 ns. After that, two parallel productive MD simulations of 6000 ns and 4000 ns long with different microscopic initial conditions carried out in the NPT ensemble, respectively. The most representative structure was identified by the cluster analysis^31^ over the equilibrated trajectories (after 1000 ns). To assess the convergence of the simulated trajectory, we considered both the time evolution of backbone atoms' root-mean-square deviation (RMSD, see Fig. S2†) of PDI and their projections on the top essential dynamical spaces obtained from a standard covariance analysis. Following Hess's criterion,^40^ these projections were next compared with those expected for a random reference. The observed negligible overlap (*i.e.* cosine content close to 0, data not shown) confirms *a posteriori* adequate sampling of the complex conformations around the equilibrium position. Hydrogen bond interactions were defined to be present if the atomic distance between the acceptor and donor atoms is below 3.5 Å and the angle among the hydrogen-donor–acceptor atoms is below 30 degree. Hydrophobic interactions were defined to be present if the center-of-mass distance between side chains are smaller than 4.5 Å.^41^

## Results

### 1. Rutin bound and inhibited MBP-fused recombinant human and mouse PDI proteins

We first tried X-ray crystallography to define the binding of PDI with rutin. With this original structural biology goal, we generated recombinant proteins of PDI and its fragments fused with maltose binding protein (MBP). MBP is a 43 kDa protein commonly used as fusion tag to increase the crystallizability of fused protein. In addition, MBP tag augments water solubility of target proteins and facilitates expression of recombinant proteins.^42^ For human PDI, we constructed MBP-PDI-b′x and MBP-PDI-b′xa′ recombinant proteins. All proteins were expressed in *E. coli* and purified by Ni-NTA chromatography followed by gel filtration chromatography (Fig. 1C and D).

**Fig. 2:**
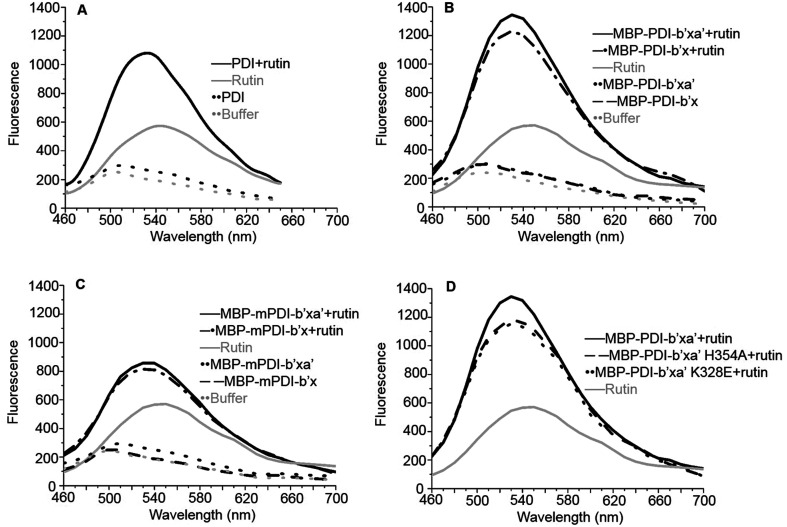
Fluorescence binding assays of rutin to the full-length human PDI (A), human MBP-PDI-b′x/b′xa′ (B), murine MBP-mPDI-b′x/b′xa′ (C) and human MBP-PDI-b′xa′ mutants (D). Rutin alone [gray solid line] had maximum emission at 550 nm. Assay buffer [gray dot line] had little fluorescence. (A) The full-length human PDI [black dot line], (B) MBP-PDI-b′x [black dash line] and MBP-PDI-b′xa′ [black dot line], (C) MBP-mPDI-b′x [black dash line] and MBP-mPDI-b′xa′ [black dot line], had little fluorescence alone. While (A) PDI:rutin complex [black solid line], (B) MBP-PDI-b′x:rutin complex [black dash-dot line] and MBP-PDI-b′xa′:rutin complex [black solid line], (C) MBP-mPDI-b′x:rutin complex [black dash-dot line] and MBP-mPDI-b′xa′:rutin complex [black solid line] (D) MBP-PDI-b′xa′ (black solid line) and its mutants, K328E (black dot line) and H354A (black dash line) compound with rutin, have enhancement in fluorescence when excitation at 430 nm and the maximum emission wavelength move to 530 nm.

**Fig. 3:**
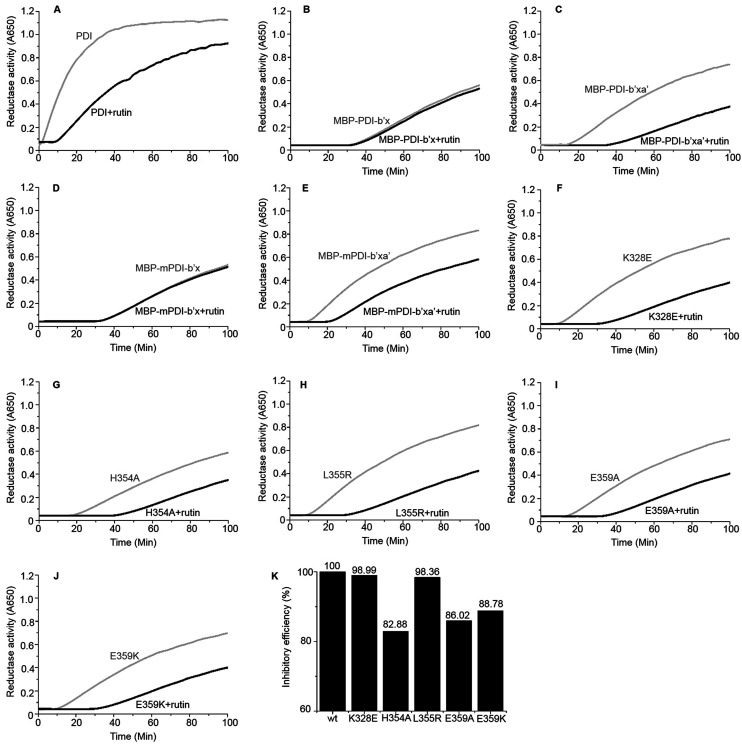
Reductase activity of PDI and MBP-fused PDI proteins in the absence (gray) and presence (black) of rutin (100 μM). (A) Full-length human PDI; (B) human MBP-PDI-b′x; (C) human MBP-PDI-b′xa′; (D) murine MBP-mPDI-b′x; (E) murine MBP-mPDI-b′xa′; (F) K328E; (G) H354A; (H) L355R; (I) E359A; (J) E359K. (K) The inhibitory efficiency of rutin to wt MBP-PDI-b′xa′ and its mutants at 100 min after addition of rutin were analyzed by the increase of turbidity (OD_650_).

We performed additional experiments to study if these MBP-fused recombinant proteins can bind to rutin and have reductase functions. We previously developed a direct binding assay of rutin to PDI.^21^ Rutin emits fluorescence when excited at 430 nm alone and has a maximal emission peak at 550 nm, while human PDI has little fluorescence excited at 430 nm alone. With addition of PDI into rutin, the fluorescence intensity of the ligand significantly increased and the wavelength of peak emission appeared blue-shifted to 530 nm (Fig. 2A). Our results for MBP-fusion proteins showed that rutin indeed bound to MBP-PDI-b′x and MBP-PDI-b′xa′ fusion proteins (Fig. 2B). The IC_50_ of these MBP-fusion proteins are similar with that of the full-length human PDI (Fig. S3†). As a negative control to rule out the possibility of MBP interaction with rutin, we measured the fluorescence of rutin in the presence of three other different MBP-fusion proteins, and found no change of fluorescence at all (Fig. S4†). We also found the reductase activities of MBP-PDI-b′xa′ through insulin reduction assays (Fig. 3C). Taken together, these results demonstrated that MBP fusion did not perturb PDI function and rutin binding, which is not surprising as MBP is a compact protein and usually does not affect the activity of target proteins.

Next, we studied the molecular interaction of PDI from murine origin (mPDI) with rutin. Although the effect of rutin was demonstrated in a murine thrombosis model,^14,20^ it is unknown whether rutin can inhibit mPDI directly. We first generated MBP-mPDI-b′x and MBP-mPDI-b′xa′ recombinant proteins and purified them to high homogeneity (Fig. 1C). We then carried out direct binding assays, and found that rutin bound to mPDI (Fig. 2C). The binding affinities of murine proteins (IC_50_ of about 30 μM) were similar to those of human counterparts (Fig. S3†). The reductase activity of murine MBP-mPDI-b′xa′ was also comparable to that of the human counterparts (Fig. 3C and E). Thus, mPDI possessed similar characteristics of human PDI in interacting with rutin.

Furthermore, we constructed a number of MBP-PDI-b′xa′ proteins with site-directed mutations on K328, H354, L355 and E359 (see below for the design of these mutants). These mutants were also expressed and purified by the methods described above (Fig. S5†).

### 2. A molecular model of PDI:rutin complex revealed by *in silico* simulations

We originally wanted to crystalize the MBP-fused PDI or mPDI or their fragments in complex with rutin in order to understand the molecular interaction of rutin with PDI. Unfortunately, we did not obtain any diffracting crystals despite thousands of crystallization trials, presumably due to the weak interaction between PDI and rutin.

**Fig. 4:**
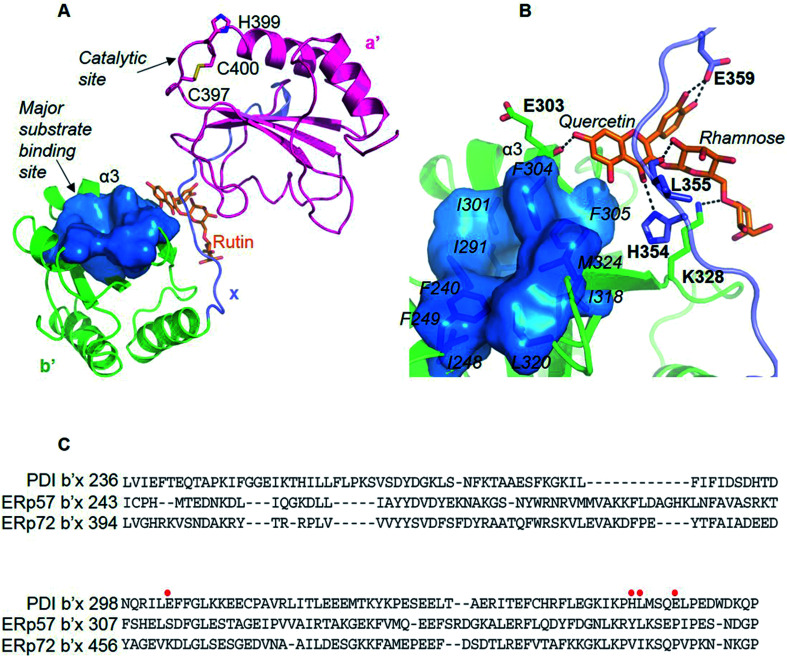
The molecular model of PDI bound to rutin obtained from docking and MD studies. (A) Rutin binding pose in the binding interface between b′ and x linkers. The substrate-binding site in b′ domain was highlighted by blue surface. (B) The interaction patterns between rutin and PDI. Hydrogen bonds were indicated with black dashed lines. (C) Sequence alignment of b′x domains from members of PDI family known to be secreted from platelets or endothelial cells. The residues involved in the interactions with rutin (E303, H354, L355 and E359) were highlighted by red dots.

We then resorted to *in silico* methods to generate a molecular model through molecular docking calculations and molecular dynamics (MD) simulations based on the available crystal structure of PDI (PDB ID: 4EL1 ^26^). Both the current studies using MBP-fused PDI and previous direct binding assays^21^ showed that the b′ domain of PDI is the major binding site of rutin. Our docking and MD studies also revealed that rutin indeed bound to PDI mainly at the b′ domain, but also contacted with the x linker (Fig. 4A). In our structural model, the quercetin moiety of rutin formed stable hydrogen bonds with the backbone oxygen atom of E303 from the helix α3 of b′ domain and with the side-chains of H354 and E359 from x linker (Fig. 4B, quercetin site). The stabilities of such intermolecular interactions were supported by the analysis of the occupancies of formation and average distances of hydrogen bonds across MD simulations (Table S1†), as well as by the mutagenesis studies (see following section). Moreover, one additional stable hydrogen bond was observed between the hydroxyl group at position 2 of the glucose of rutin and the backbone oxygen atom of L355 (Fig. 4B and Table S1†).

In contrast to the stable interactions established between PDI and the quercetin and glucose moieties of rutin, the putative hydrogen bonds contributed by the rhamnose moiety of rutin and PDI residues (K328, E330 and K352, named rhamnose site) were non-optimal and transient across the MD simulations, as indicated by the relatively low occupancies of formation of hydrogen bonds and large standard deviations of the average distances between the donor and acceptor atoms (Table S1†). To further assess the effect of the rhamnose moiety of rutin on binding to PDI, we performed *in silico*-based ADME analysis on both rutin and isoquercetin (Table S2†). The results showed that the omission of this ring may not apparently change the ADME properties of rutin. Therefore, the stability analysis of the putative interactions between PDI and rutin suggested that quercetin glycosylated at 3 position with glucose may be the major contributor to the binding affinity, while the rhamnose moiety may not contribute significantly.

### 3. Validation of PDI:rutin complex model by site-directed mutagenesis

To validate the binding site of rutin on PDI predicted by *in silico* simulations, we constructed a list of mutants of human MBP-PDI-b′xa′ on the basis of the structural model (Fig. 4): K328E, H354A, L355R, E359A, and E359K. Among them, H354A and E359A mutations were intended to verify the quercetin binding site on PDI. Other mutations, including the introduction of charges to hydrophobic residue or charge-reversal mutation, drastically changed amino acid properties to induce stronger effect of the mutations.

We first used the fluorescence-based binding assays to measure the direct binding of rutin to the mutants. When added to rutin, mutant proteins like K328E and H354A showed similar fluorescence profiles. The fluorescence intensities of K328E and H354A mutants were also similar to that of the wild-type (wt) MBP-PDI-b′xa′ protein (Fig. 2D). These results showed that our fluorescence-based direct binding assay was not able to distinguish the differences introduced by the mutations, reflecting the low sensitivity of this direct binding assay.

**Fig. 5:**
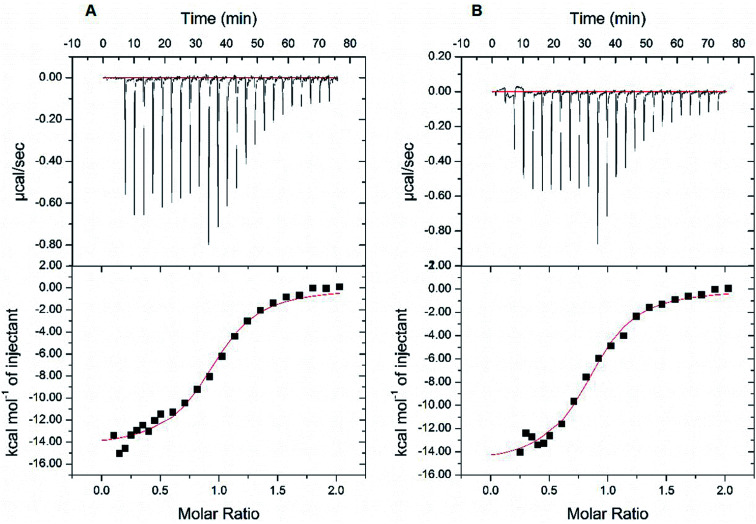
Isothermal titration calorimetry (ITC) measurements of the binding affinity between wt MBP-PDI-b′xa′ and rutin. The binding affinity of wt MBP-PDI-b′xa′ to rutin was measured by ITC method twice. With the modification of the volume of titration of rutin, we fitted the S-shape ITC curves with both upper and lower platforms. The data collected in the two parallel assays (A and B) were repeatable: the dissociation constants were 2.31 μM and 2.39 μM, the stoichiometric ratios were 0.94 and 0.86, and Δ*G* were −7.73 kcal mol^−1^ and −7.71 kcal mol^−1^, respectively.

Next, we resorted to a more sensitive method, isothermal titration calorimetry (ITC), to measure the direct binding. Due to the weak interaction, this method requires large amounts of high-purity protein samples (about 240 μL at 20–50 μM) to be titrated by the highest concentration of rutin (its water solubility of about 200 μM), so that the release of heat as a result of complex formation reached plateau phase rapidly and was enough to be measured. A problem we encountered is that the starting phase of the S-shape curve of heat measurement cannot always be resolved using the typical titration program of ITC, thus affecting the accuracy of dissociation constant (*K*_d_) measurement from the S-shape ITC curve. This was due to the use of equal volume of small molecule titrant (∼2.5 μL) used in the typical ITC titration. Therefore, we established an optimized ITC titration scheme by using variable drop sizes, which started with small drops (1 μL at first ten times of injection) and ended with large drops (2 μL). We thus obtained S-shape ITC curves with well-defined upper and lower platforms (Fig. 5 and S6†), which allowed the accurate measurements of *K*_d_ values.

At first, we carried out this improved ITC method to measure the *K*_d_ of rutin to wt MBP-PDI-b′xa′. In two independent ITC experiments, the *K*_d_ was found to be 2.39 μM and 2.31 μM, respectively (Fig. 5). This result validated that our improved ITC procedure is quite sensitive and demonstrated that the ITC results were reproducible. We then applied this ITC method for rutin binding to wt MBP-PDI-b′xa′ and its mutants (Table 1 and Fig. S6†). The results showed that the stoichiometric ratio for rutin binding to wt MBP-PDI-b′xa′ protein was about 1 : 1, which was consistent with our previously reported measurements of the full-length human PDI^21^ and further confirmed that the binding site of rutin was indeed within the b′xa′ domains of PDI. In general, the effect of mutation on rutin binding to PDI was modest (Table 1), but detectable in these measurements. The mutant used to probe the rhamnose site of rutin, K328E, demonstrated almost the same binding affinity compared with wt MBP-PDI-b′xa′ (2.39 *vs.* 2.29 μM, Table 1), indicating that this residue did not contribute significantly to the binding of rutin. Other mutations (H354A, L355R, E359A, and E359K) had effects to different degrees on the rutin binding (*K*_d_ of 5.98, 3.50, 4.10, 4.48 μM, respectively). Notably, the binding of rutin to the H354A mutant (*K*_d_ of 5.98 μM) decreased by about 3 times compared with wt MBP-PDI-b′xa′ protein (*K*_d_ of 2.39 μM).

**Table 1:** Dissociation constant (*K*_d_) and stoichiometry ratio (*N*) of recombinant MBP-PDI-b′xa′ and its mutants to rutin. Site-directed mutagenesis of MBP-fused human PDI proteins were designed based on *in silico* simulations of rutin bound to human PDI. The interactions between rutin and MBP-PDI-b′xa′ proteins were measured with isothermal titration calorimetry (ITC) method. Data were expressed as mean ± S.D

	*N*	*K*_d_ (μM)	Δ*G* (kcal mol^−1^)
wt	0.86 ± 0.02	2.39 ± 0.45	−7.71 ± 0.48
K328E	0.86 ± 0.02	2.29 ± 0.36	−7.72 ± 0.31
H354A	0.72 ± 0.03	5.98 ± 1.08	−7.15 ± 0.86
L355R	1.93 ± 0.03	3.50 ± 0.39	−7.46 ± 0.23
E359A	1.18 ± 0.03	4.10 ± 0.74	−7.39 ± 0.49
E359K	1.21 ± 0.02	4.48 ± 0.58	−7.31 ± 0.45

### 4. Measurements of reductase activities of the site-directed mutants further validated the PDI:rutin complex model

We further used the insulin reduction assay to measure the reductase activities of these site-directed MBP-PDI-b′xa′ mutants. This assay has typically good reproducibility as shown by a control experiment with three repetitions (Fig. S7†). We found the PDI mutants maintained reductase activities in the presence of dithiothreitol (DTT). The H354A mutation lowered the reductase activity of wt MBP-PDI-b′xa′ (Fig. 3G) while other mutants had similar reductase activities with that of wt MBP-PDI-b′xa′ (Fig. 3C, F and H–J). Addition of rutin inhibited the reductase activities of both wt MBP-PDI-b′xa′ and the mutants (Fig. 3C and F–J). As control, we showed that rutin and other other rutin analogues (see section below) did not perturb the insulin reductase assay (Fig. S8†). We quantified the inhibition of rutin on these MBP-PDI-b′xa′ mutants by measuring the increase of turbidity (OD_650_ at 100 min) upon addition of rutin (Fig. 3K). We found that, compared with the wt MBP-PDI-b′xa′, the inhibitory efficiency of rutin on H354A, E359A and E359K decreased about 20% while L355R showed modest decrease. Together, these results are in agreement with our *in silico* simulations and ITC assays, and supported that the residues H354 and E359 are important in the binding of PDI to rutin.

### 5. Inhibitory activities of rutin analogues against PDI

**Fig. 6:**
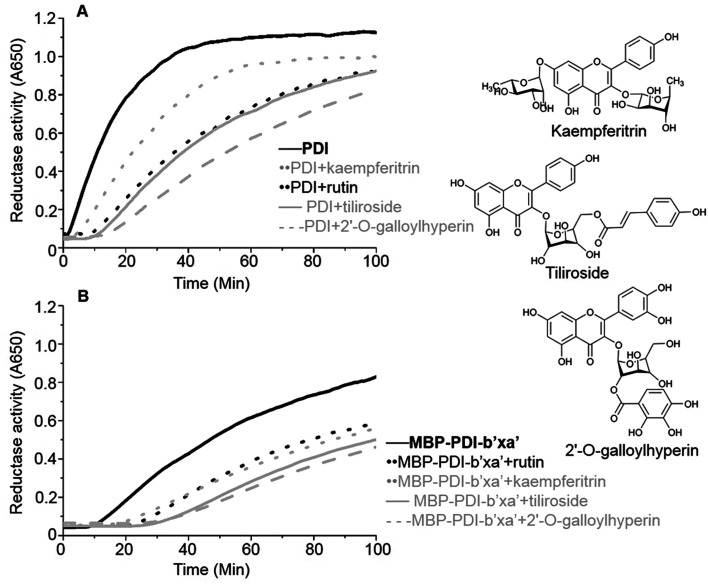
PDI inhibitory activities of three analogues of rutin. The inhibitory activities of three analogues of rutin (100 μM) were measured by insulin reduction assays. Rutin (black in dot line), kaempferitrin (gray in dot line), tiliroside (gray in solid line) and 2′-*O*-galloylhyperin (gray in dash line) were added into the full-length human PDI (A) or wt MBP-PDI-b′xa′ (B). The black in solid line represents the reduction of insulin by the full-length human PDI (A) or wt MBP-PDI-b′xa′ (B) alone. The structural diagram of three analogues of rutin were showed at the right line.

We identified a series of rutin analogues and purchased three of them including kaempferitrin, tiliroside and 2′-*O*-galloylhyperin where the second sugar ring is different from rutin. Their inhibitory activities against the full-length human PDI and PDI fragment (wt MBP-PDI-b′xa′) were also measured by insulin reduction assays. The reductase activities of both PDI and MBP-PDI-b′xa′ were inhibited by these analogues as observed in the assays of rutin (Fig. 6). The inhibitory activity of kaempferitrin to the full-length human PDI was smaller than that of rutin to PDI. The results indicated that the second sugar ring at position 3 of rutin did not contribute to the binding to PDI. This was in agreement with the results obtained in the previous study of the structure–activity relationship between flavonols and their inhibitory potency against PDI, where the IC_50_ values of the inhibitors with or without the second sugar ring at 3 position were similar.^20^ This was also consistent with our *in silico* simulations and ITC assays, both of which suggested that the residue K328 involved in the interaction with the second sugar ring at position 3 of rutin did not function in the binding of rutin to PDI.

## Discussion

### 1. Protein flexibility of PDI

PDI is a dynamic protein with two redox-dependent conformations clearly identified by crystal structural studies of either human^26,43^ or yeast PDI.^44^ In the crystal structure of the oxidized fungal PDI b′xa′ bound to its substrate, α-synuclein, the protein took an open conformation with an exposed major substrate binding site in b′ domain bound to the substrate. At the reduced condition, there was no direct binding of the substrate, α-synuclein, presumably due to the closed conformation.^45^ Recent MD studies confirmed such redox-regulation of PDI conformations, but revealed a much larger degree of flexibility.^46,47^ Small-angle X-ray scattering (SAXS) experiments,^48,49^ including ours,^21^ were also carried out to study the solution conformation of PDI. All these results suggest a hypothesis of redox-regulated substrate binding of PDI where PDI is in an open conformation in the oxidized state (in the presence of GSSG) to expose its hydrophobic substrate binding pocket, and becomes compact in the reduced form to expel the substrate away from the substrate binding site.

In our current study, we used the reducing condition in all assays because the insulin reduction assay was carried out in the presence of DTT, and the recombinant protein contains two free cysteines and needs reducing condition to maintain its activities. We additionally did an experiment to study if the redox state will affect the binding site of rutin. We found that the oxidative condition (GSSG) gave a higher fluorescence signal compared to the reducing condition (DTT) in the direct binding assay of rutin to wild type PDI (Fig. S9†). For PDI H354 mutant, such redox-induced fluorescence enhancement has a magnitude identical to wt PDI (Fig. S9†). This indicated that the binding sites of rutin on PDI most likely not affected by the redox state of PDI. This is understandable because the binding site locates far away from the free cysteine residues, which are most like one key sensing elements for the redox state (GSSG/DTT).

There are only limited numbers of crystal structures of the full-length human PDI, presumably reflecting its dynamic nature and the difficulty of PDI crystallization. The resolved crystal structures showed that the four domains of human PDI arranged in a horseshoe shape, wherein the a and a′ domains faced each other at the open end of the horseshoe while b and b′ domains formed its closed end (Fig. 1B). The structure of PDI in oxidized and reduced states also showed that the b′xa′ region of PDI was mainly responsible for the redox-regulation of its conformations.^26^ A tryptophan residue of the x linker (20 amino acids long) bound to the substrate binding site,^50^ demonstrating one example of conformational flexibility induced by the x linker.

We narrowed down the rutin binding site to the b′xa′ fragment of PDI, and used this fragment and its fusion with MBP for the co-crystallization with rutin in order to reduce conformational entropy penalty and increase the crystallizability. However, we still were unable to obtain diffracting crystals of rutin in complex with MBP-fused recombinant PDI fragments despite extensive efforts, likely due to the weak nature of the complex. Thus, we resorted to thorough computational methods to predict the binding model of rutin bound to PDI. In light of relatively low binding affinity of rutin to PDI, we use two carefully designed assays to validate the binding model. Both assays consistently showed that the H354A mutation lowered the binding affinity of PDI fragment to rutin and the activity of PDI fragment. We did not combine the single mutants together for further validation. A better approach will be nuclear magnetic resonance (NMR) for further validation of the PDI binding residues of rutin, but such method requires major effort.

### 2. Structural basis of PDI interacting with rutin

All four domains of PDI participate in the substrate recognition,^51–53^ and the non-catalytic domains, especially the b′ domain, are the major substrate binding site.^54^ The PDI b′ domain contains a characteristic thioredoxin-like fold containing five β-sheet strands and four α-helices. The major substrate binding site was formed between the α1 and α3 helices and constituted of conserved amino acids (F240, F249, I301, F304, F305, L320 and M324) (Fig. 4B). This binding site is largely hydrophobic in nature, and appears not to be the potential binding site of rutin because of the amphipathic nature of rutin structure.

We previously demonstrated that the b′x domain of PDI is the primary binding site of rutin.^21^ Our current *in silico* study and site-directed mutagenesis showed that the binding site was uniquely located at the interface between b′ and x linkers. The residues H354, L355 and E359 from the b′x domain were key for rutin binding to PDI (Table 1 and Fig. 4B). Notably, these key residues were not conserved across the extracellular thiol isomerases, indicating a candidate interface to target PDI specifically (Fig. 4C). This was consistent with previous experimental data that rutin did not inhibit other thiol isomerases, including ERp5, ERp57, ERp72 or thioredoxin.^20^

Our model also showed that the second sugar ring at 3 position of rutin was not important for binding to PDI. Three analogues of rutin which mainly differ on the second sugar ring at 3 position showed similar inhibitory activities against the full-length human PDI and wt MBP-PDI-b′xa′ (Fig. 6). In agreement with this, mutation on its binding residue (K328E) had no effect on the binding of PDI (Table 1 and Fig. 3F).

The structural model of rutin binding of PDI may drive a new strategy for structural optimization of rutin-based PDI inhibitors by increasing their binding affinities to PDI. For example, it is valuable to perform thorough scaffold hopping on the quercetin and glucose moieties of rutin. Structure-based pharmacophore strategies can be also used to identify or design novel moieties targeting PDI rhamnose site with enhanced stabilities.

### 3. Potential mechanism of rutin inhibition on the redox activities of PDI

Rutin binds and inhibits PDI selectively and reversibly. According to our current PDI:rutin model, the binding sites of rutin on PDI were primarily located at the interface of b′ domain and x linker of PDI. Rutin bound near the major substrate binding site of PDI, but did not directly bind to the major substrate binding site (Fig. 4). However, rutin was not too far away from the opening of substrate binding site (4.3 Å to residue F304). Thus, rutin may perturb the conformation of the major substrate binding site, and inhibit substrate binding to PDI. On the other hand, rutin binding at the x linker may hinder the flexibility of PDI, which is critical for the catalytic activity of PDI. In addition, our MD simulations also showed that rutin induced a closed conformation of PDI, blocking the accessibility of the substrate binding site (Fig. S10†). Collectively, it appears that it is the allosteric effect of rutin that is mainly responsible for inhibiting the redox activities of PDI.

## Conclusion

Rutin demonstrated effective anticoagulant activity in human by inhibiting PDI activity. However, the rutin does not have good bioavailability and high dose is required. In fact, rutin inhibits PDI activity with an IC_50_ at micromolar range. Identification of molecular interaction between rutin and PDI is critical for further improvement of pharmacological profile of rutin. We developed a strategy to study such weak interaction combining computational and experimental methods. The results demonstrate the feasibility of the current strategy. In addition, rutin shows a unique binding site right next to the canonical major substrate binding site located at the b′ domain. Thus, this work provides the structural basis for the inhibitory mechanism of rutin to PDI, and will be useful for further development of potent inhibitors with higher binding affinity to PDI for therapeutic applications.

## Conflicts of interest

There are no conflicts to declare.

## Abbreviations

PDI: Protein disulfide isomerase
MD: Molecular dynamics
ITC: Isothermal titration calorimetry
K328E: Lysine328Glutamicacid
H354A: Histidine354Alanine
L355R: Leucine355Arginine
E359A: Glutamicacid359Alanine
E359K: Glutamicacid359Lysine
ER: Endoplasmic reticulum
nM: Nanomole L^−1^
μM: Micromole L^−1^
mM: Millimole L^−1^
mg: Milligram
kg: Kilogram
MBP: Maltose binding protein
HPLC: High performance liquid chromatography
LIC: Ligation independent clone
PCR: Polymerase chain reaction
IPTG: Isopropyl-β-d-thiogalactoside
EDTA: Ethylenediaminetetraacetic acid
β-ME: β-Mercaptoethanol
μL: Microliter
nm: Nanometre
DTT: Dithiothreitol
s: Second
GAFF: General AMBER force field
PME: Particle Mesh-Ewald
RMSD: Root-mean-square deviation
WT: Wild type
*K*_d_
: Dissociation constant
NMR: Nuclear magnetic resonance
E303: Glutamicacid303
F240, F249, F304, F305: Phenylalanine240, 249, 304,305
I301: Isoleucine301
L320: Leucine320
M324: Methionine324
SDS-PAGE: Sodium dodecyl sulfate-polyacrylamide gel electrophoresis
ADME: Absorption, distribution, metabolism and excretion
GSSG: Oxidized glutathione
SAXS: Small-angle X-ray scattering

## Supplementary Material

RA-008-C8RA02683A-s001
