# Supplementary material for: Molecular basis of rutin inhibition of protein disulfide isomerase (PDI) by combined in silico and experimental methods
Source: RSC Adv. 2018 May 21;8(33):18480–91. doi: 10.1039/c8ra02683a (PMC9080521; doi:10.1039/c8ra02683a)
Supplement: RA-008-C8RA02683A-s001 [file RA-008-C8RA02683A-s001.pdf]

Electronic Supplementary Material (ESI) for RSC Advances.
This journal is © The Royal Society of Chemistry 2018

Electronic Supplementary Information

Molecular basis of rutin inhibition of protein disulfide isomerase (PDI) by combined *in silico* and experimental methods

Xu Wang[a,b] , Guangpu Xue[c], Meiru Song[c], Peng Xu[a], Dan Chen[c], Cai Yuan[d], Lin Lin[e], Robert Flaumenhaft[e], Jinyu Li[*c], Mingdong Huang[*a,c]

[a] State Key Laboratory of Structural Chemistry, Fujian Institute of Research on the Structure of Matter, Chinese Academy of Sciences, Fuzhou 350002, China
[b] College of Life Science, Fujian Normal University, Fuzhou 350117, China
[c] College of Chemistry, Fuzhou University, Fuzhou 350116, China
[d] College of Biological Science and Engineering, Fuzhou University, Fuzhou 350116, China
[e] Beth Israel Deaconess Medical Center, Harvard Medical School, 330 Brookline Ave., Boston, MA, USA
[*] Corresponding authors: Jinyu Li: Email: j.li@fzu.edu.cn; Tel. +86 188 60103237;
Mingdong Huang: Email: HMD_lab@fzu.edu.cn; Tel. +86 138 59021285.

Supplementary Methods

1. The purity of small-molecule chemicals was analysed by HPLC (Fig. S1). We used Welch Xtimade C18 chromatographic column (size: 150 mm X 4.6 mm) for analysis.

	**Concentration (µM)**	**Mobile phase**	**Detection wavelength (nm)**	**Injection volume (µL)**	**Flow rate (ml/min)**	**Column pressure (MPa)**
**Rutin**	200	$CH_3OH:H_2O$(1% HOAc)=32:68	257	20	1	13
**Kaempferitrin**	500	$CH_3CN:H_2O$(0.3%H_3PO_4)=30:70	345	20	1	10
**Tiliroside**	700	$CH_3CN:H_2O$(0.04%H_3PO_4)=15:85	254	20	1	13
**2’-O-galloylhyperin**	330	$CH_3CN:H_2O$(0.2% H_3PO_4)=19:81	258	20	1	12

2. Binding affinity (IC_{50}) of rutin to human or murine MBP-fused PDI proteins

The MBP-fused PDI proteins (18 µM) were incubated with a gradient of 10 rutin’s concentrations differed by 2- or 3-fold and 3 repetitions in 384-well black plate at room temperature for 30 min before measurements. The total assay volume was filled to 100 µL using the assay buffer (pH 7.4) contains 150 mM NaCl, 20 mM Tris, 1 mM EDTA, 1 mM β-ME and 5% glycerin. The fluorescence emission spectrum of the mixture were measured with the excitation at 430 nm and the emission at 530 nm at room temperature on a BioTek Synergy microplate reader with a sensitivity of 100. The data were analyzed by GraphPad Prism (Fig. S3).

Supplementary Figures

Fig. S1 Purity analysis by HPLC of small-molecule chemicals used in the experimental assays. The purity of rutin, kaempferitrin, tiliroside and 2’-O-galloylhyperin were 98.75%, 94.84%, 86.02% and 96.23%, respectively.

A. Rutin (98.75%)

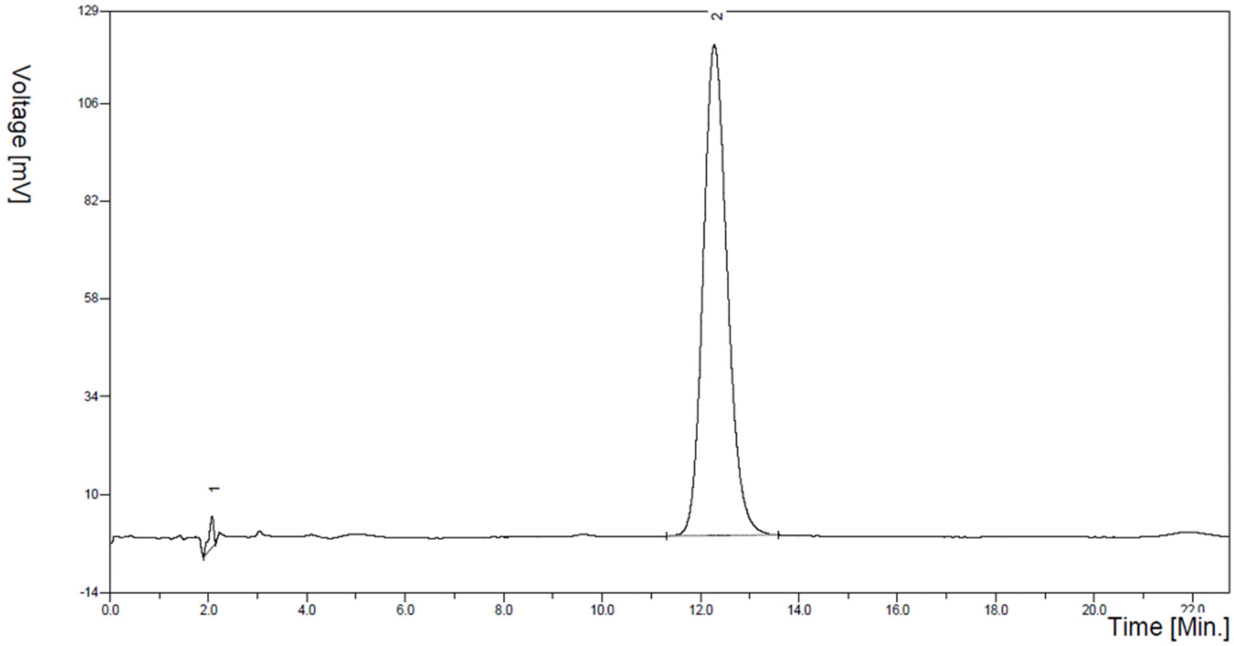

Number	Retention time (Min)	Height (mV)	Area (mv. sec)	Percentage (%)
1	2.07	7.54	51.81	1.2464
2	12.28	120.59	4104.57	98.7536

B. Kaempferitrin (94.84%)

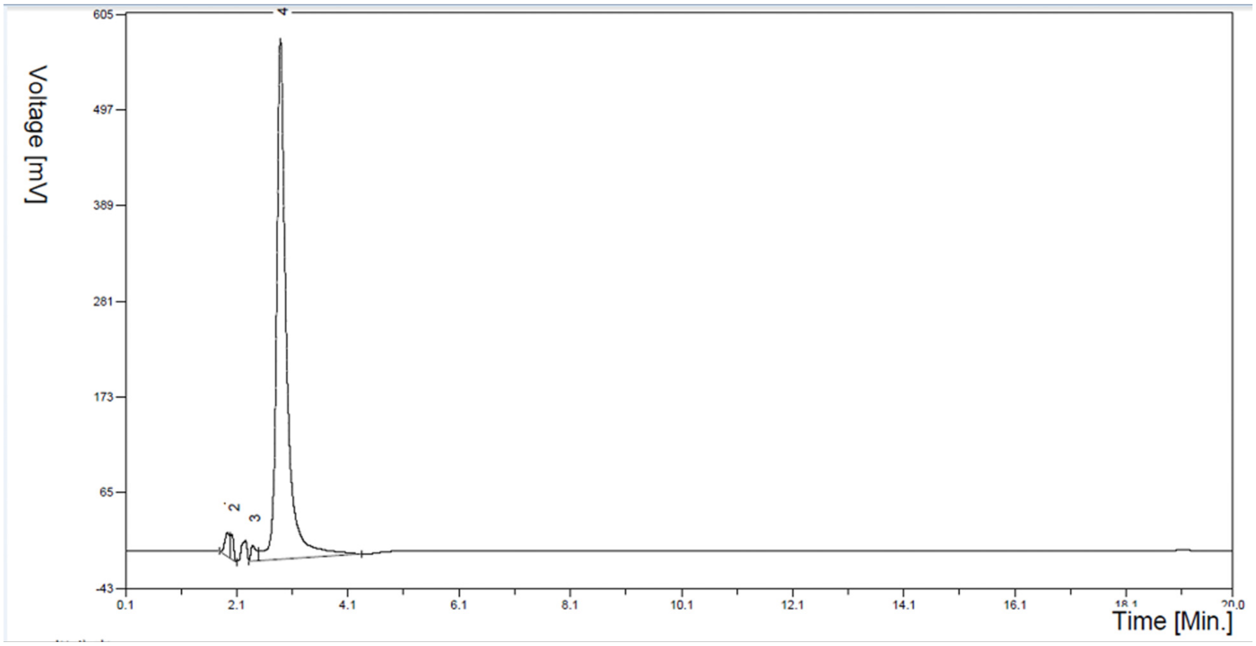

Number	Retention time (Min)	Height (mV)	Area (mv. sec)	Percentage (%)
1	1.93	27.25	165.60	2.1367
2	2.01	27.60	112.03	1.4455
3	2.39	17.50	122.28	1.5778
4	2.89	587.15	7350.46	94.8400

C. Tiliroside (86.02%)

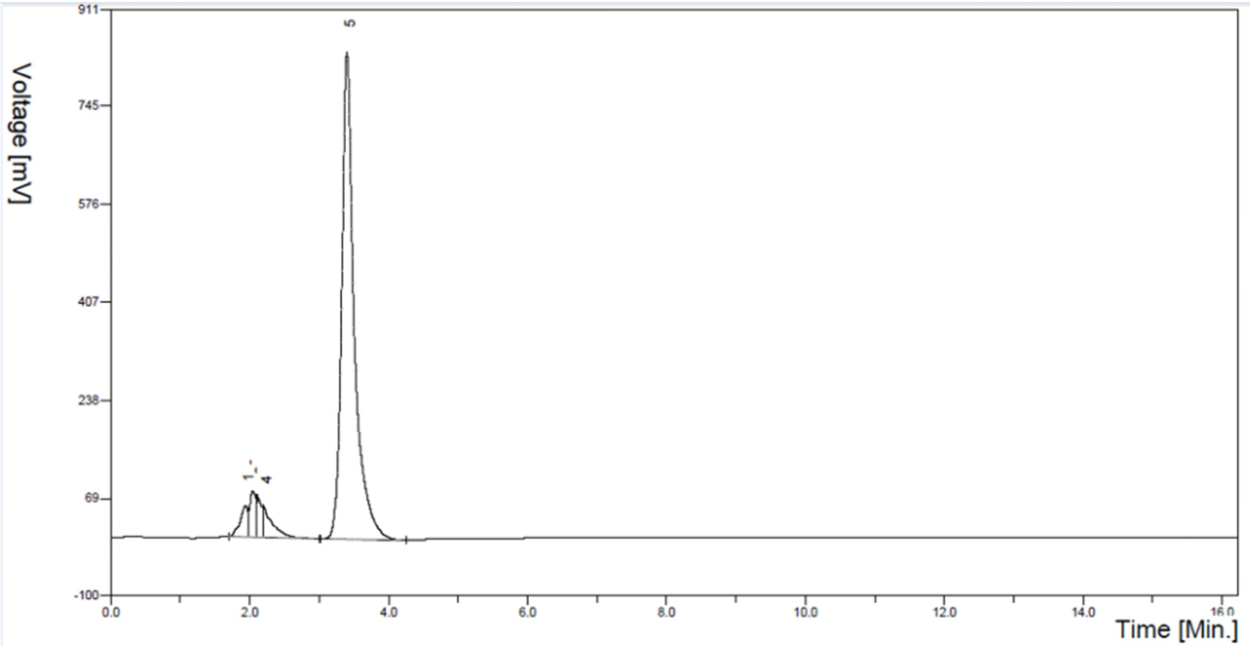

Number	Retention time (Min)	Height (mV)	Area (mv. sec)	Percentage (%)
1	1.94	54.35	417.73	3.3970
2	2.04	80.41	493.16	4.0103
3	2.12	67.40	299.97	2.4393
4	2.20	51.89	508.59	4.1359
5	3.40	839.19	10577.70	86.0175

D. 2’-O-galloylhyperin (96.23%)

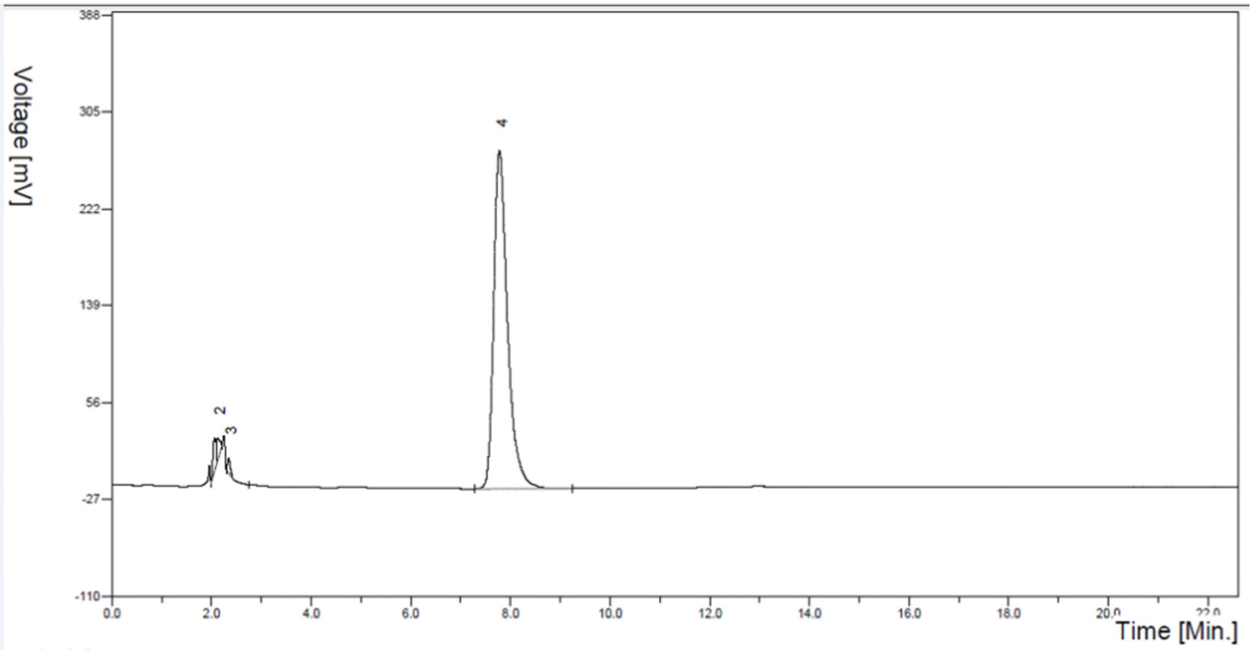

Number	Retention time (Min)	Height (mV)	Area (mv. sec)	Percentage (%)
1	2.06	29.02	107.94	1.8549
2	2.12	19.04	92.25	1.5853
3	2.34	11.22	19.22	0.3303
4	7.78	289.38	5599.72	96.2295

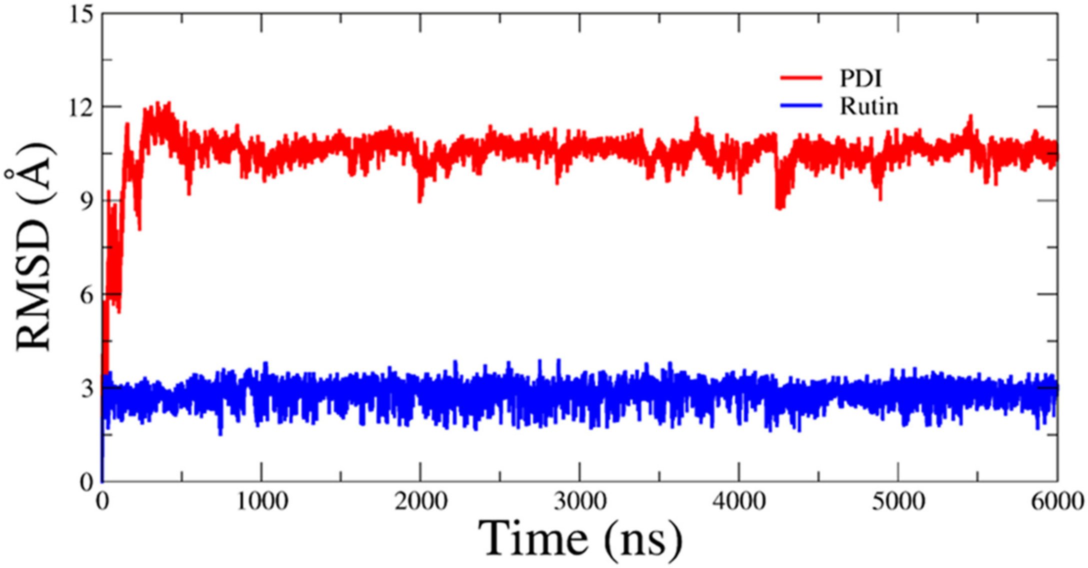

Fig. S2. Root-mean-square deviation (RMSD) of backbone atoms of PDI (red) and heavy atoms of rutin relative to the initial structures as a function of MD simulation time.

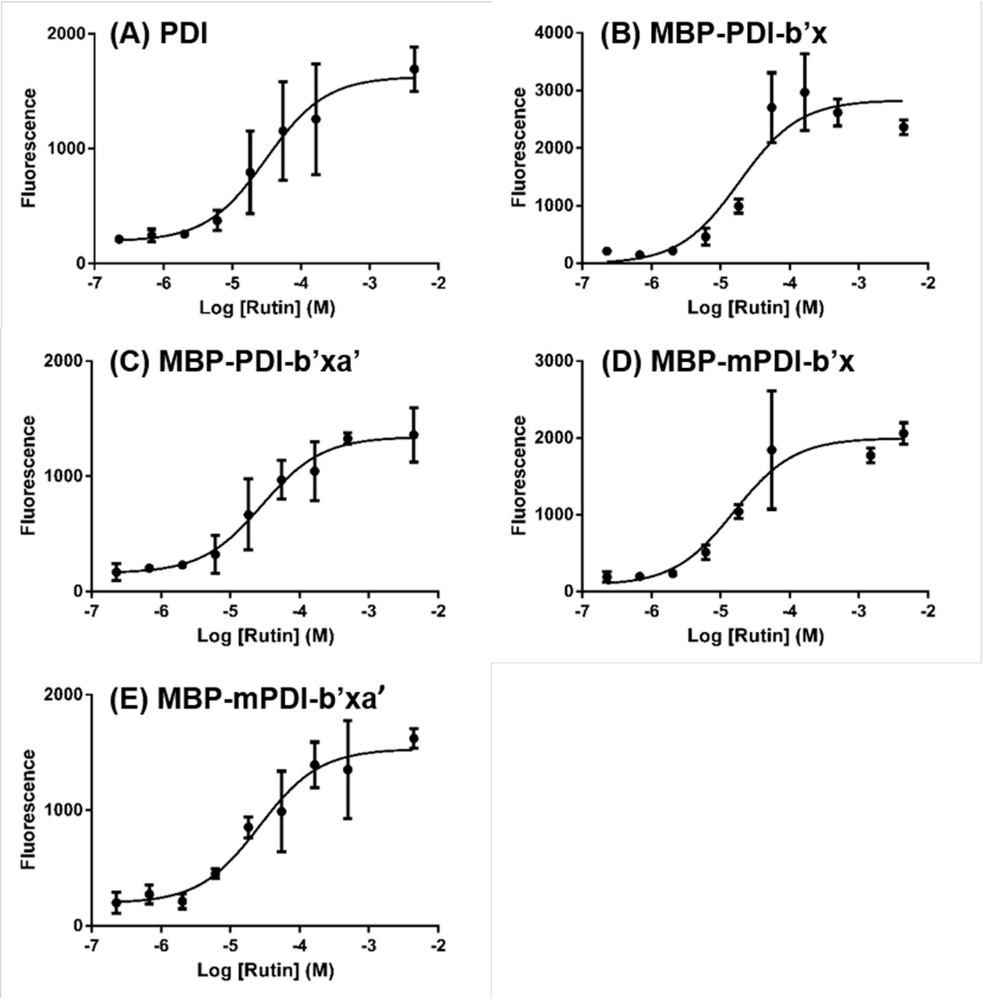

Fig. S3. Fluorescence assay on the interactions between rutin and human or murine MBP-fused PDI proteins. All these proteins have similar binding affinity to rutin. A-E: IC_{50} values for the full-length human PDI and MBP-fused PDI proteins binding to rutin were: (A) full-length human PDI, 31.1 μM; (B) human MBP-PDI-b'x, 18.7 μM; (C) human MBP-PDI-b'xa', 28.8 μM; (D) murine MBP-mPDI-b'x, 15.7 μM; (E) murine MBP-mPDI-b'xa', 26.3 μM. *Error bas* indicate ±S.E, n=3. Standard Error: (A) the full-length human PDI, 0.18 μM; (B) human MBP-PDI-b'x, 0.16 μM; (C) the human MBP-PDI-b'xa', 0.14 μM; (D) the murine MBP-mPDI-b'x, 0.15 μM; (E) the murine MBP-mPDI-b'xa', 0.15 μM.

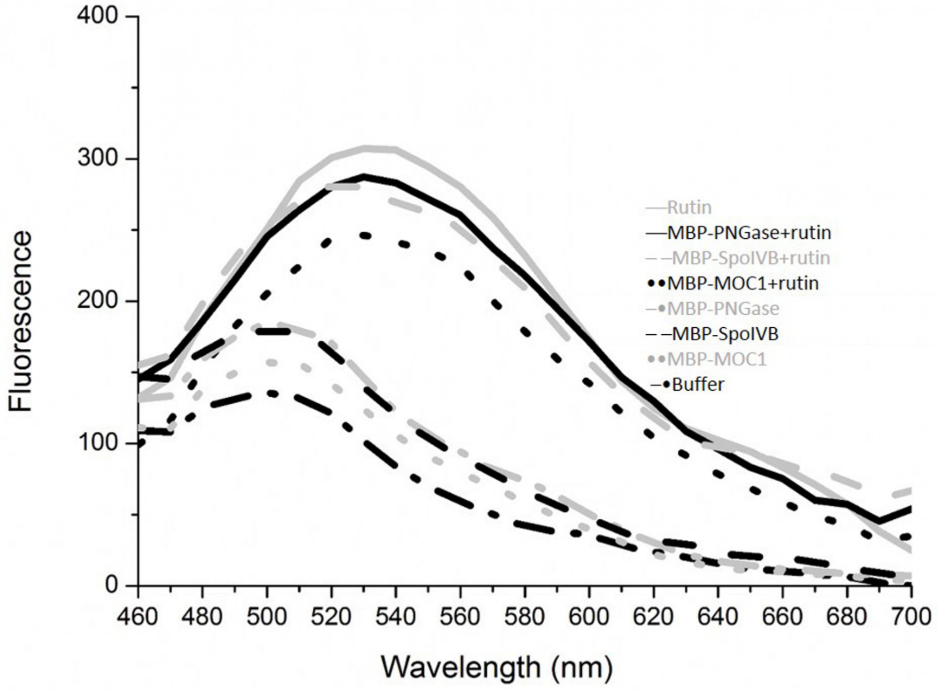

Fig. S4. Rutin does not bind to MBP as shown by fluorescence measurements of rutin in the presence of three different MBP-fusion proteins. The MBP-fusion proteins (MBP-PNGase protein (gray dash-dot line), MBP-SpoIVB protein (gray dash line) and MBP-MOC1 protein (gray dot line) at 18 μM did not have fluorescence compared to the assay buffer with excitation at 430 nm. Addition of rutin (55 μM) to these proteins (MBP-PNGase in black solid line, MBP-SpoIVB in black dash line and MBP-MOC1 in black dot line) gave the signals almost identical to rutin itself (gray solid), which has a maximum fluorescence at 550 nm.

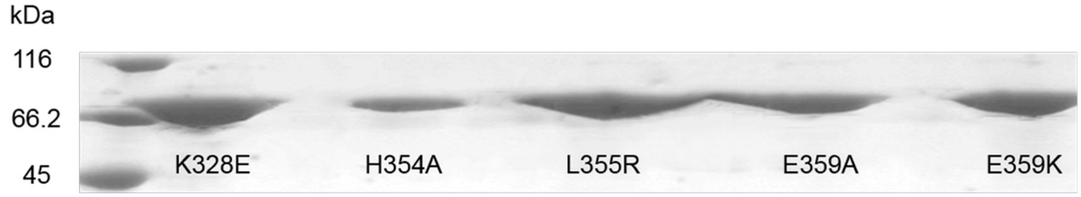

Fig. S5. The reduced SDS-PAGE analysis of the purified MBP-PDI-b'xa' mutants. The molecular weight of K328E, H354A, L355R, E359A and E359K are 71.8 kDa, 71.7 kDa, 71.9 kDa, 71.8 kDa and 71.8 kDa, respectively.

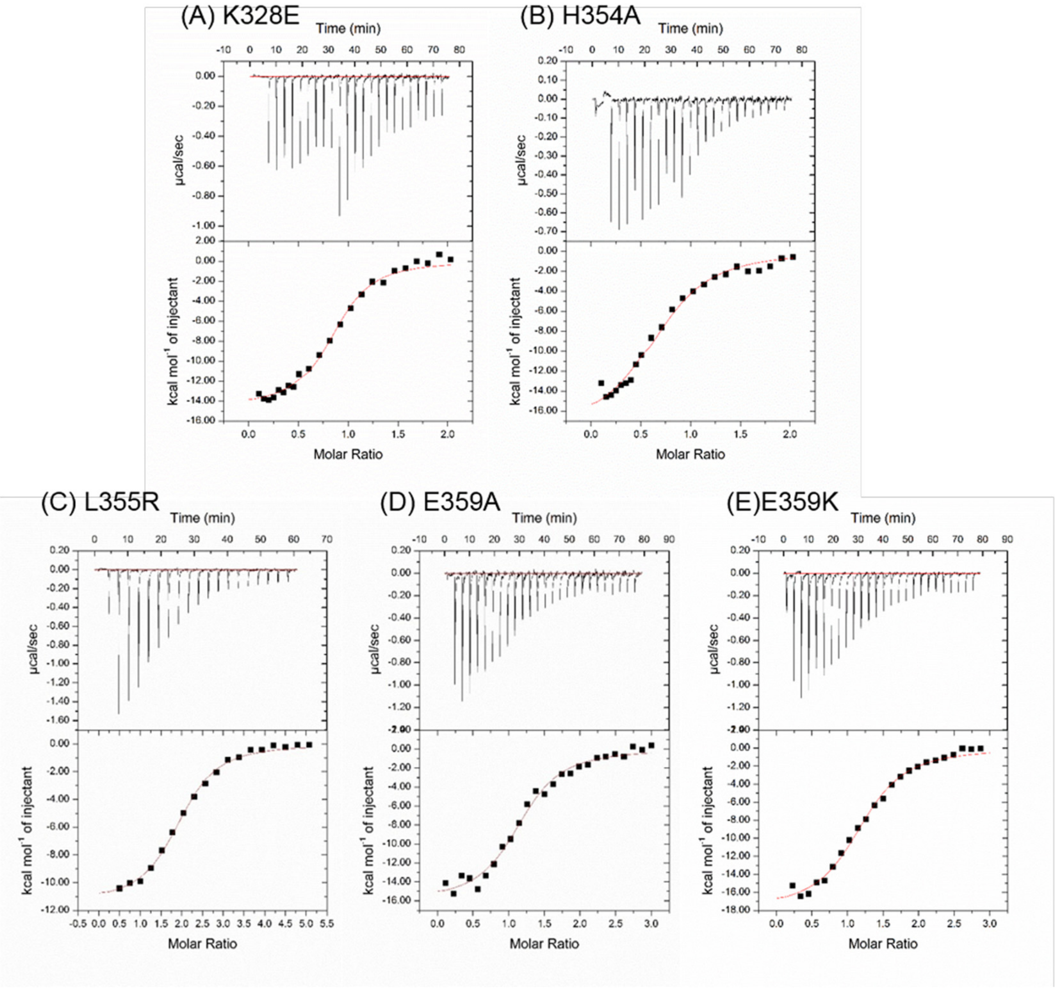

Fig. S6. Isothermal titration calorimetry (ITC) measurements of the binding affinity between the mutants of wt MBP-PDI-b'xa' and rutin. The interactions of the mutants of wt MBP-PDI-b'xa' to rutin was measured by ITC method. (A) K328E; (B) H354A; (C) L355R; (D) E359A; (E)E359K.

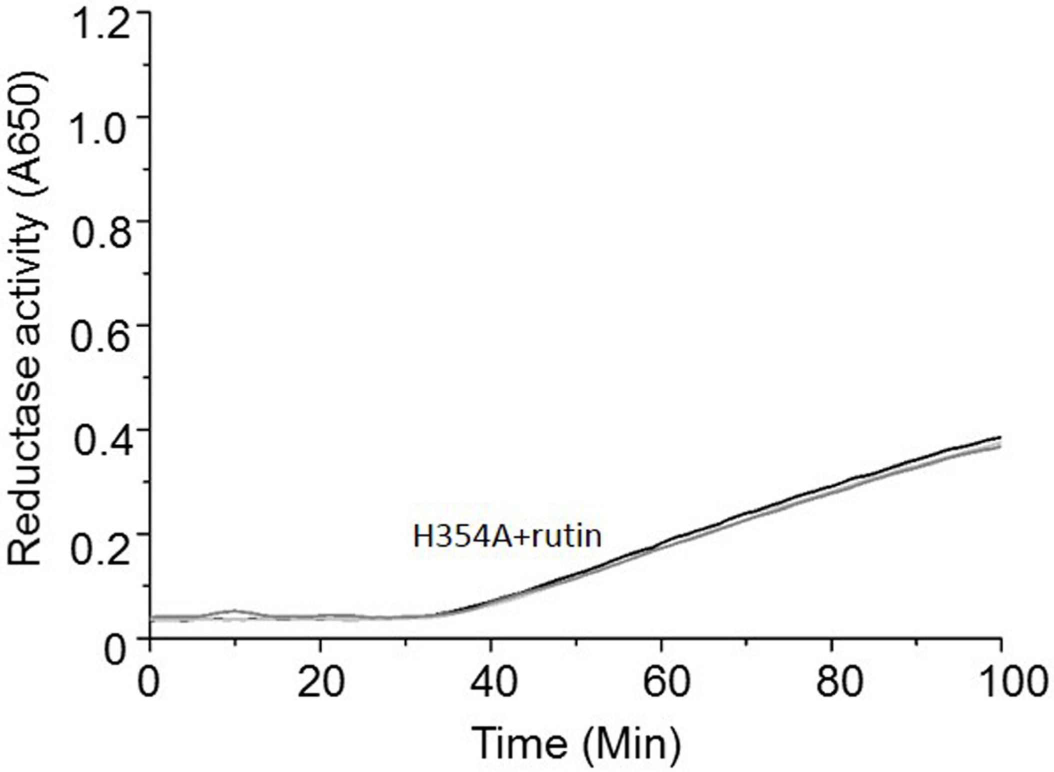

Fig. S7. Insulin reductase assay has good reproducibility as shown by three independent experiments of rutin (100 μM) inhibition on the insulin reduction catalyzed by MBP-PDI-b'xa'-H354A mutant.

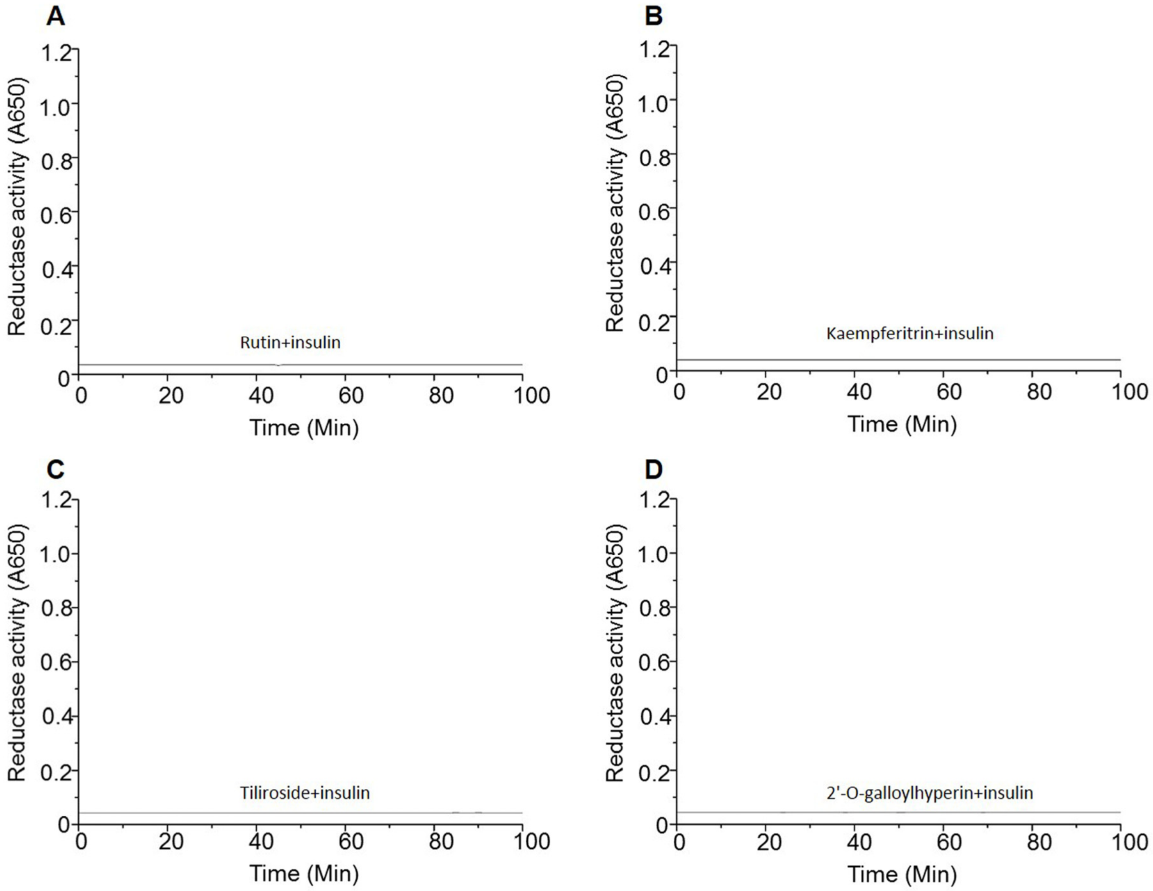

Fig. S8. Small molecular inhibitors did not perturb insulin reduction assay. 100 µM of rutin **(A)**, kaempferitrin **(B)**, tiliroside **(C)** and 2'-O-galloylhyperin **(D)** were added into the assay solution in the absence of any PDI or its fragments.

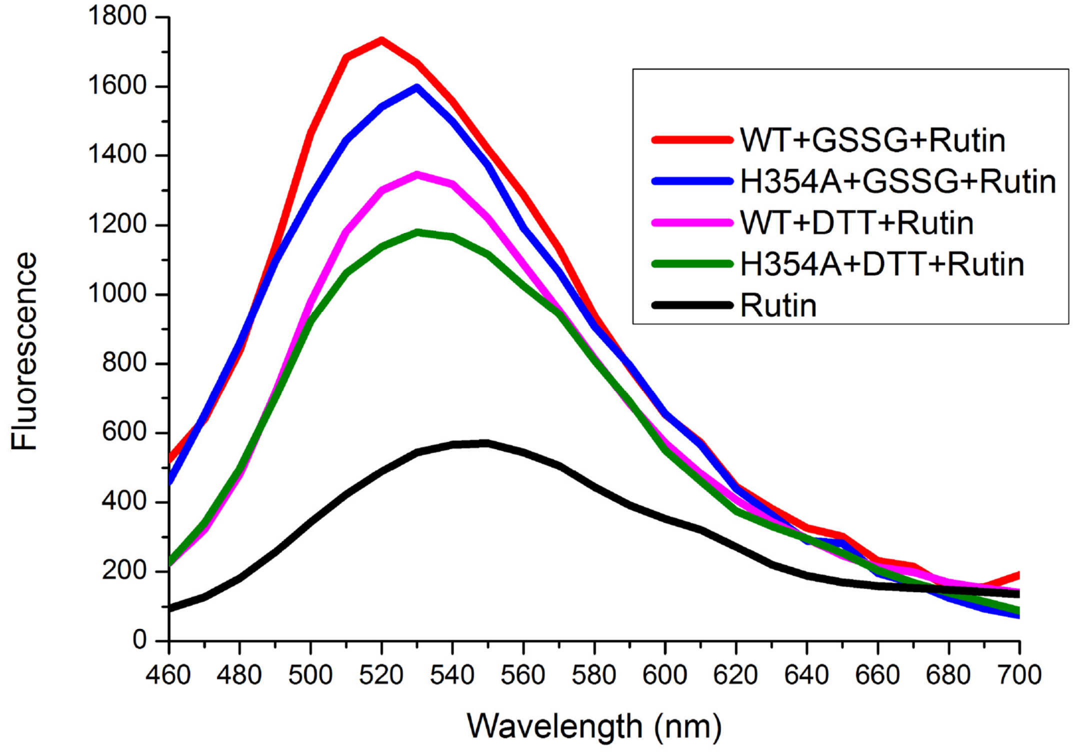

Fig. S9. Rutin binding to PDI (MBP-PDI-b'xa') is sensitive to GSSH/DTT ratio, but not to the mutation of H354A as shown by fluorescence-based binding assay at reducing (1 mM DTT) or oxidative (5 mM GSSG) condition.

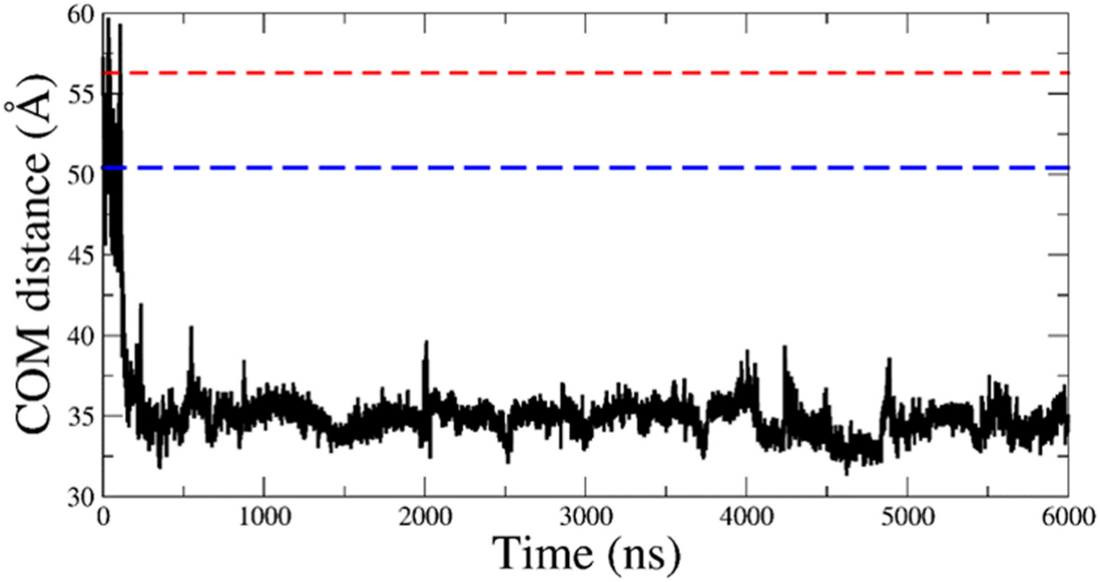

Fig. S10. The center-of-mass (COM) distance between a and a' domains as a function of MD simulation time. The corresponding distances in the crystal structures of PDI in the oxidized (PDB ID: 4EL1) and reduced states (PDB ID: 4EKZ)[1] were displayed by red and blue dashed lines.

References

1. C. Wang, W. Li, J. Q. Ren, J. Q. Fang, H. M. Ke, W. M. Gong, W. Feng and C. C. Wang, *Antioxid. Redox Signaling*, 2013, **19**, 44-53.

Supplementary Tables

Table S1. Hydrogen bond interactions between rutin and PDI.

Rutin	PDI	Distance (Å)[a]	Occupancy (%)[b]
O@7-OH	O@E303	3.2 ± 0.5	89.6
O@4'-OH	$O_{\varepsilon 1}$@E359	3.4 ± 0.7	74.0
O@5'-OH	$O_{\varepsilon 1}$@E359	3.4 ± 0.5	77.2
O@4-ketone	N_{δ}@H354	2.9 ± 0.3	98.8
O@2-OH	O@L355	2.7 ± 0.1	99.8
O@1-ether	N_{ξ}@K328	4.1 ± 1.1	40.0
O@3-OH	O@K352	3.8 ± 0.8	44.8
O@4-OH	$O_{\varepsilon 1}$@E330	7.2 ± 2.8	1.6
O@5-OH	$O_{\varepsilon 2}$@E330	6.8 ± 1.4	2.8

[a] Average atomic distances between hydrogen bond donor and acceptor calculated on the equilibrium trajectory of MD simulations.

[b] Occupancy of the formation of hydrogen bonds across the equilibrium trajectory of MD simulations.

Table S2. Chemical properties of rutin and isoquercetin.

Terms listed in the table (from left to right): partition coefficient (logP) predicted by Molinspiration (http://www.molinspiration.com/) (logPm) and ALOGPS2.1 (logPa), [1] aqueous solubility (AS) in g/L predicted by ALOGPS2.1, [1] drug-like score (Score) predicted by OSIRIS [2] and molecular weight (MW) in Da.

	logPm[a]	logPa[a]	AS[b]	Score[c]	MW
Rutin	-1.06	-0.14	0.16	1.93	610.52
Isoquercetin	-0.36	0.02	0.14	1.66	464.38

[a] The value of logP is defined as the logarithm of the ratio of the concentrations of compound between octanol and water. It is a measurement of the lipophilicity of compounds and is used for drug absorption prediction. From the magnitude of the logP of a compound, one can infer its ease of transport through the cell membrane. According to the rule of 5 by Lipinski, [3] compounds with logP greater than 5 are more likely a poor absorption or permeation.

[b] AS governs both the rate of dissolution of the compound and the maximum concentration of compound reached in the gastrointestinal fluid. [4]

[c] The drug-like score predicted by OSIRIS[2] is calculated with the summation of score values of substructure fragments that are present in the compound under investigation. The distributions of drug-like score values calculated from 15000 commercially available chemicals and 3300 traded drugs show that about 80% of the drugs have a positive drug-like score values while the big majority of chemicals accounts for the negative values. [2]

References

1. I. V. Tetko and G. I. Poda, *J. Med. Chem.*, 2004, **47**, 5601-5604.
2. T. Sander, J. Freyss, M. von Korff, J. R. Reich and C. Rufener, *J. Chem. Inf. Model.*, 2009, **49**, 232-246.
3. C. A. Lipinski, F. Lombardo, B. W. Dominy and P. J. Feeney, *Adv. Drug Delivery Rev.*, 2012, **64**, 4-17.
4. J. Wang and T. Hou, *Comb. Chem. High Throughput Screening*, 2011, **14**, 328-338.

Supplementary Data

1. Raw experimental data of Fig. 2

1) Fig. 2A:

Wavelength	buffer	PDI	rutin	PDI+rutin
460	105	139	91	134
462	113	154	99	144
464	108	158	99	171
466	119	171	111	189
468	123	163	126	183
470	121	178	136	212
472	137	182	132	251
474	151	187	152	264
476	149	201	166	285
478	164	204	158	324
480	160	190	176	342
482	163	202	186	400
484	189	229	221	411
486	180	245	225	454
488	182	230	251	509
490	207	239	253	534
492	225	240	278	596
494	226	258	305	610
496	237	272	338	679
498	238	271	345	716
500	260	283	351	751
502	268	294	370	789
504	243	309	411	867
506	249	306	435	875
508	262	297	425	915
510	256	298	417	952
512	237	295	467	988
514	237	289	460	996
516	230	294	470	1001
518	224	291	484	1014
520	206	280	492	1041
522	227	276	513	1086
524	237	289	517	1071
526	208	276	507	1084
528	196	268	553	1050

530	193	268	545	1075
532	194	272	550	1079
534	213	251	555	1043
536	196	246	550	1092
538	182	258	564	1107
540	201	261	570	1059
542	198	243	583	1060
544	182	253	580	1049
546	200	240	596	994
548	188	256	563	988
550	164	245	562	946
552	167	240	561	922
554	179	228	549	905
556	155	224	535	908
558	168	231	548	877
560	161	228	558	849
562	175	225	529	853
564	158	214	525	835
566	165	204	516	828
568	149	213	507	783
570	158	203	493	725
572	140	197	492	703
574	138	184	476	715
576	154	176	448	682
578	130	174	468	666
580	135	157	457	637
582	133	180	428	618
584	124	172	405	552
586	117	175	413	561
588	115	174	406	535
590	117	163	382	529
592	110	156	360	497
594	117	152	372	493
596	123	145	356	462
598	112	116	360	488
600	97	124	351	458
602	89	131	340	420
604	101	139	327	410
606	105	122	319	398
608	111	100	309	399
610	87	121	330	402

612	102	124	317	350
614	93	110	277	361
616	72	105	285	323
618	76	108	259	317
620	74	99	267	274
622	65	99	255	284
624	71	95	232	272
626	72	86	223	296
628	59	93	232	257
630	58	99	218	250
632	64	76	215	249
634	72	76	221	246
636	51	72	217	251
638	67	70	212	225
640	63	74	191	239
642	76	85	174	226
644	56	66	174	217
646	58	80	185	205
648	59	68	187	177
650	51	61	167	183

2) Fig. 2B

**Wavelength**	**MBP-PDI-b'x+ rutin**	**MBP-PDI-b'xa'+ rutin**	**rutin**	**buffer**	**MBP-PDI-b 'xa'**	**MBP-PDI-b'x**
460	256	226	91	108	158	171
470	345	323	136	151	187	201
480	487	474	176	189	229	245
490	684	710	253	226	258	272
500	946	983	351	243	309	306
510	1103	1194	417	230	294	294
520	1196	1288	492	237	289	276
530	1233	1350	545	213	251	246
540	1201	1325	570	182	253	240
550	1122	1213	562	179	228	224
560	1008	1093	558	158	214	214
570	889	947	493	138	184	176
580	776	824	457	124	172	175
590	666	679	382	117	152	145
600	549	570	351	101	139	122

610	471	489	330	93	110	105
620	410	402	267	71	95	86
630	337	347	218	64	76	72
640	282	294	191	56	66	80
650	282	250	167	48	58	76
660	230	207	160	40	55	66
670	227	196	154	36	50	60
680	211	187	148	28	46	55
690	128	120	142	22	42	53
700	121	153	136	20	35	46

3) Fig. 2C

**Wavelength**	**MBP-mPDI-b'xa'+rutin**	**MBP-mPDI-b'x+rutin**	**rutin**	**buffer**	**MBP-mPDI-b'xa'**	**MBP-mPDI-b'x**
460	207	218	91	113	154	119
470	266	294	136	137	182	149
480	346	368	176	163	202	180
490	464	471	253	225	240	237
500	646	631	351	268	294	249
510	763	744	417	237	295	230
520	824	785	492	227	276	208
530	851	818	545	194	272	196
540	865	811	570	198	243	200
550	810	773	562	167	240	155
560	731	708	558	175	225	165
570	670	638	493	140	197	154
580	573	556	457	133	180	117
590	494	471	382	110	156	123
600	409	401	351	89	131	105
610	361	370	330	102	124	93
620	304	302	267	65	99	72
630	254	257	218	64	76	51
640	221	219	191	76	85	58
650	184	212	167	68	83	56
660	180	155	160	65	80	53
670	133	142	154	50	76	52
680	134	144	148	48	72	48
690	124	95	142	50	70	44
700	89	86	136	45	66	40

4) Fig. 2D

Wavelength	rutin	MBP-PDI-b'xa'+rutin	K328E	H354A
460	91	226	219	220
470	136	323	359	366
480	176	474	449	461
490	253	710	705	717
500	351	983	900	926
510	417	1194	1038	1064
520	492	1288	1126	1137
530	545	1350	1139	1173
540	570	1325	1151	1184
550	562	1213	1065	1098
560	558	1093	996	1041
570	493	947	890	924
580	457	824	815	841
590	382	679	624	653
600	351	570	548	584
610	330	489	409	434
620	267	402	390	391
630	218	347	347	324
640	191	294	275	293
650	167	250	243	268
660	160	207	186	190
670	154	196	175	170
680	148	187	168	152
690	142	120	105	95
700	136	153	90	96

2. Raw data of Fig. 3

Kinetic read	MBP-PDI	MBP-mPDI	MBP-PDI	MBP-mPDI	MBP-PDI	MBP-mPDI	MBP-PDI	MBP-mPDI
	-b'x	-b'x	-b'xa'	-b'xa'	-b'x	-b'x	-b'xa'	-b'xa'
					+rutin	+rutin	+rutin	+rutin
0:00:32	0.041	0.043	0.042	0.042	0.042	0.041	0.047	0.042
0:01:32	0.042	0.044	0.042	0.043	0.043	0.043	0.047	0.042

0:02:32	0.042	0.044	0.042	0.043	0.042	0.043	0.048	0.042
0:03:32	0.043	0.046	0.042	0.043	0.043	0.046	0.048	0.043
0:04:32	0.042	0.045	0.042	0.044	0.043	0.045	0.048	0.043
0:05:32	0.042	0.045	0.043	0.044	0.044	0.044	0.048	0.043
0:06:32	0.042	0.046	0.043	0.044	0.044	0.044	0.049	0.044
0:07:32	0.042	0.046	0.043	0.048	0.043	0.044	0.048	0.044
0:08:32	0.042	0.046	0.043	0.044	0.043	0.044	0.048	0.044
0:09:32	0.042	0.046	0.046	0.045	0.043	0.044	0.048	0.043
0:10:32	0.042	0.045	0.053	0.048	0.043	0.045	0.049	0.043
0:11:32	0.043	0.046	0.062	0.056	0.043	0.045	0.048	0.043
0:12:32	0.042	0.045	0.073	0.067	0.043	0.045	0.048	0.043
0:13:32	0.042	0.046	0.085	0.08	0.043	0.045	0.048	0.043
0:14:32	0.042	0.045	0.098	0.095	0.043	0.045	0.049	0.043
0:15:32	0.042	0.046	0.112	0.11	0.043	0.045	0.049	0.043
0:16:32	0.042	0.045	0.125	0.125	0.042	0.045	0.049	0.043
0:17:32	0.042	0.047	0.138	0.14	0.042	0.045	0.048	0.043
0:18:32	0.042	0.046	0.153	0.156	0.042	0.045	0.049	0.043
0:19:32	0.042	0.045	0.167	0.17	0.042	0.044	0.049	0.044
0:20:32	0.042	0.045	0.182	0.186	0.042	0.045	0.049	0.044
0:21:32	0.042	0.045	0.197	0.2	0.042	0.044	0.051	0.046
0:22:	0.042	0.045	0.21	0.216	0.042	0.044	0.055	0.05

32								
0:23:32	0.042	0.045	0.225	0.233	0.042	0.044	0.06	0.056
0:24:32	0.043	0.045	0.239	0.247	0.043	0.045	0.067	0.063
0:25:32	0.042	0.045	0.254	0.261	0.043	0.045	0.073	0.071
0:26:32	0.042	0.046	0.268	0.275	0.042	0.045	0.081	0.079
0:27:32	0.042	0.046	0.282	0.289	0.042	0.044	0.088	0.088
0:28:32	0.042	0.046	0.297	0.305	0.042	0.045	0.096	0.097
0:29:32	0.042	0.046	0.307	0.317	0.043	0.044	0.105	0.106
0:30:32	0.043	0.046	0.323	0.332	0.042	0.046	0.114	0.116
0:31:32	0.043	0.046	0.337	0.343	0.043	0.045	0.123	0.126
0:32:32	0.043	0.047	0.348	0.356	0.045	0.046	0.133	0.136
0:33:32	0.045	0.049	0.36	0.369	0.049	0.049	0.141	0.146
0:34:32	0.048	0.053	0.369	0.384	0.054	0.053	0.151	0.156
0:35:32	0.053	0.058	0.377	0.394	0.06	0.059	0.162	0.165
0:36:32	0.058	0.064	0.39	0.405	0.067	0.065	0.172	0.177
0:37:32	0.064	0.069	0.402	0.414	0.072	0.071	0.181	0.187
0:38:32	0.07	0.076	0.404	0.424	0.079	0.078	0.192	0.197
0:39:32	0.076	0.083	0.417	0.436	0.086	0.084	0.202	0.207
0:40:32	0.083	0.091	0.426	0.448	0.094	0.092	0.212	0.216
0:41:32	0.089	0.097	0.442	0.459	0.101	0.099	0.222	0.225
0:42:32	0.097	0.105	0.442	0.467	0.11	0.107	0.232	0.236

0:43:32	0.104	0.114	0.462	0.479	0.117	0.115	0.242	0.244
0:44:32	0.112	0.121	0.472	0.486	0.126	0.122	0.252	0.254
0:45:32	0.12	0.13	0.479	0.492	0.135	0.132	0.262	0.263
0:46:32	0.128	0.138	0.491	0.502	0.144	0.139	0.272	0.274
0:47:32	0.136	0.147	0.497	0.512	0.153	0.148	0.281	0.282
0:48:32	0.144	0.155	0.516	0.524	0.161	0.157	0.288	0.288
0:49:32	0.152	0.163	0.525	0.532	0.171	0.165	0.299	0.297
0:50:32	0.161	0.173	0.535	0.54	0.179	0.173	0.307	0.305
0:51:32	0.17	0.182	0.537	0.548	0.187	0.182	0.319	0.316
0:52:32	0.179	0.19	0.546	0.556	0.196	0.191	0.324	0.319
0:53:32	0.187	0.2	0.562	0.566	0.206	0.2	0.33	0.328
0:54:32	0.196	0.208	0.57	0.565	0.216	0.208	0.336	0.337
0:55:32	0.205	0.217	0.579	0.582	0.226	0.219	0.345	0.343
0:56:32	0.215	0.228	0.58	0.599	0.234	0.226	0.355	0.35
0:57:32	0.223	0.237	0.6	0.606	0.243	0.235	0.361	0.357
0:58:32	0.233	0.245	0.602	0.606	0.252	0.244	0.371	0.365
0:59:32	0.241	0.254	0.609	0.614	0.261	0.252	0.378	0.373
1:00:32	0.25	0.262	0.619	0.623	0.271	0.261	0.387	0.376
1:01:32	0.256	0.271	0.619	0.634	0.279	0.269	0.396	0.384
1:02:32	0.264	0.28	0.629	0.634	0.288	0.277	0.403	0.387
1:03:	0.274	0.288	0.632	0.639	0.296	0.285	0.409	0.396

32								
1:04:32	0.286	0.297	0.645	0.649	0.306	0.293	0.413	0.402
1:05:32	0.297	0.304	0.643	0.656	0.312	0.301	0.424	0.411
1:06:32	0.304	0.313	0.654	0.663	0.321	0.309	0.43	0.418
1:07:32	0.312	0.32	0.662	0.672	0.329	0.316	0.433	0.422
1:08:32	0.322	0.329	0.666	0.677	0.34	0.325	0.44	0.426
1:09:32	0.331	0.336	0.669	0.684	0.349	0.333	0.446	0.438
1:10:32	0.337	0.344	0.683	0.692	0.358	0.344	0.457	0.44
1:11:32	0.344	0.354	0.675	0.693	0.368	0.346	0.463	0.45
1:12:32	0.35	0.36	0.691	0.707	0.377	0.353	0.466	0.451
1:13:32	0.36	0.368	0.698	0.707	0.383	0.359	0.469	0.456
1:14:32	0.364	0.373	0.7	0.716	0.39	0.365	0.475	0.462
1:15:32	0.373	0.382	0.71	0.716	0.398	0.373	0.48	0.466
1:16:32	0.38	0.389	0.715	0.722	0.407	0.38	0.484	0.474
1:17:32	0.388	0.396	0.72	0.729	0.411	0.386	0.495	0.479
1:18:32	0.391	0.402	0.722	0.741	0.421	0.393	0.495	0.486
1:19:32	0.404	0.409	0.727	0.746	0.425	0.399	0.502	0.491
1:20:32	0.406	0.416	0.746	0.746	0.433	0.405	0.502	0.494
1:21:32	0.419	0.421	0.734	0.745	0.438	0.413	0.519	0.496
1:22:32	0.423	0.429	0.741	0.756	0.445	0.418	0.513	0.502
1:23:32	0.43	0.435	0.739	0.761	0.454	0.424	0.516	0.505

1:24:32	0.435	0.442	0.756	0.767	0.463	0.431	0.528	0.507
1:25:32	0.444	0.446	0.759	0.767	0.472	0.439	0.536	0.516
1:26:32	0.454	0.453	0.758	0.783	0.478	0.441	0.538	0.52
1:27:32	0.461	0.464	0.759	0.77	0.486	0.448	0.538	0.523
1:28:32	0.465	0.465	0.783	0.79	0.491	0.453	0.544	0.532
1:29:32	0.47	0.473	0.781	0.79	0.498	0.457	0.548	0.535
1:30:32	0.477	0.478	0.779	0.791	0.506	0.461	0.549	0.54
1:31:32	0.482	0.482	0.796	0.797	0.511	0.467	0.553	0.54
1:32:32	0.491	0.486	0.787	0.804	0.518	0.471	0.554	0.547
1:33:32	0.496	0.494	0.804	0.806	0.522	0.48	0.558	0.551
1:34:32	0.497	0.497	0.805	0.81	0.523	0.482	0.564	0.557
1:35:32	0.503	0.503	0.803	0.811	0.529	0.49	0.574	0.557
1:36:32	0.508	0.508	0.801	0.821	0.537	0.494	0.57	0.563
1:37:32	0.514	0.515	0.811	0.824	0.544	0.498	0.577	0.572
1:38:32	0.52	0.519	0.808	0.824	0.551	0.505	0.579	0.577
1:39:32	0.527	0.525	0.831	0.83	0.554	0.507	0.58	0.579
1:40:32	0.532	0.533	0.829	0.832	0.561	0.515	0.585	0.583

**Kinetic read**	**PDI**	**PDI+rutin**	**K328E**	**K328E+rutin**	**H354A**	**H354A+rutin**	**L355R**	**L355R+rutin**
0:00:32	0.048	0.073	0.039	0.041	0.043	0.041	0.039	0.042
0:01:32	0.056	0.073	0.039	0.042	0.044	0.041	0.039	0.042

0:02:32	0.085	0.073	0.04	0.042	0.043	0.041	0.04	0.042
0:03:32	0.131	0.073	0.04	0.043	0.043	0.041	0.04	0.042
0:04:32	0.181	0.074	0.04	0.043	0.043	0.041	0.041	0.042
0:05:32	0.233	0.073	0.04	0.042	0.043	0.041	0.041	0.042
0:06:32	0.283	0.073	0.041	0.042	0.043	0.042	0.041	0.042
0:07:32	0.331	0.073	0.041	0.043	0.043	0.042	0.04	0.042
0:08:32	0.379	0.073	0.041	0.043	0.043	0.042	0.041	0.042
0:09:32	0.421	0.079	0.043	0.043	0.044	0.042	0.043	0.042
0:10:32	0.466	0.087	0.047	0.043	0.043	0.042	0.048	0.042
0:11:32	0.503	0.101	0.054	0.043	0.043	0.042	0.055	0.042
0:12:32	0.546	0.116	0.063	0.043	0.043	0.042	0.065	0.042
0:13:32	0.578	0.134	0.072	0.043	0.043	0.042	0.075	0.042
0:14:32	0.618	0.15	0.083	0.043	0.043	0.042	0.087	0.042
0:15:32	0.652	0.168	0.095	0.043	0.044	0.042	0.099	0.042
0:16:32	0.682	0.185	0.106	0.043	0.043	0.042	0.112	0.042
0:17:32	0.708	0.202	0.118	0.043	0.044	0.042	0.125	0.042
0:18:32	0.736	0.221	0.13	0.043	0.047	0.042	0.138	0.042
0:19:32	0.76	0.238	0.143	0.043	0.051	0.042	0.152	0.042
0:20:32	0.78	0.255	0.157	0.043	0.055	0.042	0.166	0.042
0:21:32	0.805	0.276	0.168	0.043	0.06	0.042	0.18	0.042
0:22:3	0.81	0.29	0.182	0.043	0.067	0.042	0.195	0.043

2	4							
0:23:32	0.836	0.31	0.195	0.043	0.072	0.042	0.208	0.042
0:24:32	0.859	0.325	0.207	0.043	0.079	0.042	0.221	0.042
0:25:32	0.875	0.342	0.221	0.043	0.087	0.042	0.235	0.042
0:26:32	0.889	0.361	0.233	0.043	0.094	0.042	0.248	0.042
0:27:32	0.907	0.377	0.245	0.043	0.102	0.042	0.263	0.043
0:28:32	0.913	0.394	0.259	0.043	0.11	0.042	0.275	0.043
0:29:32	0.932	0.407	0.27	0.043	0.118	0.042	0.288	0.043
0:30:32	0.946	0.424	0.284	0.043	0.126	0.042	0.304	0.045
0:31:32	0.961	0.438	0.295	0.044	0.134	0.042	0.312	0.047
0:32:32	0.975	0.45	0.305	0.046	0.143	0.041	0.326	0.05
0:33:32	0.981	0.468	0.316	0.049	0.151	0.042	0.34	0.054
0:34:32	0.99	0.478	0.326	0.052	0.16	0.042	0.352	0.058
0:35:32	1.008	0.491	0.339	0.055	0.168	0.042	0.363	0.062
0:36:32	1.016	0.508	0.353	0.059	0.176	0.042	0.373	0.066
0:37:32	1.026	0.518	0.36	0.063	0.185	0.042	0.384	0.07
0:38:32	1.032	0.534	0.372	0.067	0.194	0.042	0.394	0.075
0:39:32	1.038	0.545	0.386	0.071	0.202	0.043	0.401	0.081
0:40:32	1.044	0.556	0.394	0.076	0.21	0.044	0.416	0.085
0:41:32	1.047	0.569	0.4	0.081	0.221	0.046	0.429	0.09
0:42:32	1.048	0.58	0.409	0.086	0.225	0.049	0.438	0.096

0:43:32	1.053	0.586	0.421	0.09	0.232	0.052	0.446	0.101
0:44:32	1.064	0.597	0.43	0.097	0.241	0.056	0.448	0.107
0:45:32	1.063	0.603	0.437	0.102	0.249	0.06	0.462	0.112
0:46:32	1.073	0.605	0.449	0.107	0.257	0.064	0.47	0.118
0:47:32	1.072	0.61	0.459	0.113	0.264	0.068	0.483	0.124
0:48:32	1.077	0.619	0.468	0.118	0.274	0.072	0.49	0.13
0:49:32	1.075	0.633	0.475	0.124	0.282	0.077	0.5	0.136
0:50:32	1.082	0.659	0.481	0.131	0.289	0.081	0.508	0.143
0:51:32	1.08	0.662	0.492	0.136	0.297	0.086	0.518	0.148
0:52:32	1.086	0.671	0.501	0.143	0.305	0.09	0.521	0.154
0:53:32	1.085	0.686	0.508	0.148	0.31	0.096	0.538	0.16
0:54:32	1.091	0.691	0.518	0.155	0.32	0.101	0.548	0.167
0:55:32	1.092	0.691	0.526	0.161	0.327	0.106	0.552	0.173
0:56:32	1.088	0.7	0.535	0.166	0.335	0.112	0.567	0.18
0:57:32	1.091	0.707	0.545	0.173	0.34	0.117	0.571	0.186
0:58:32	1.096	0.717	0.549	0.177	0.348	0.123	0.581	0.192
0:59:32	1.098	0.728	0.558	0.184	0.354	0.128	0.587	0.198
1:00:32	1.099	0.731	0.565	0.19	0.361	0.134	0.6	0.205
1:01:32	1.098	0.742	0.575	0.197	0.371	0.14	0.606	0.211
1:02:32	1.102	0.752	0.584	0.203	0.377	0.146	0.608	0.217
1:03:3	1.10	0.762	0.589	0.208	0.385	0.152	0.621	0.224

2	1							
1:04:32	1.103	0.759	0.6	0.215	0.389	0.157	0.626	0.229
1:05:32	1.105	0.768	0.606	0.221	0.398	0.163	0.634	0.235
1:06:32	1.107	0.784	0.614	0.227	0.406	0.17	0.641	0.241
1:07:32	1.105	0.785	0.618	0.233	0.41	0.175	0.644	0.247
1:08:32	1.108	0.787	0.628	0.238	0.415	0.182	0.654	0.254
1:09:32	1.105	0.794	0.632	0.245	0.423	0.186	0.656	0.26
1:10:32	1.106	0.804	0.639	0.249	0.427	0.193	0.67	0.266
1:11:32	1.109	0.814	0.648	0.254	0.435	0.199	0.676	0.271
1:12:32	1.111	0.812	0.652	0.259	0.442	0.205	0.679	0.279
1:13:32	1.111	0.823	0.657	0.266	0.447	0.211	0.683	0.284
1:14:32	1.111	0.829	0.66	0.271	0.454	0.217	0.693	0.289
1:15:32	1.111	0.831	0.662	0.277	0.46	0.223	0.698	0.294
1:16:32	1.108	0.845	0.672	0.285	0.466	0.229	0.701	0.3
1:17:32	1.112	0.834	0.675	0.29	0.475	0.234	0.704	0.304
1:18:32	1.116	0.842	0.677	0.295	0.481	0.24	0.71	0.312
1:19:32	1.114	0.85	0.688	0.299	0.479	0.245	0.709	0.317
1:20:32	1.116	0.863	0.684	0.305	0.487	0.25	0.722	0.324
1:21:32	1.115	0.873	0.693	0.31	0.495	0.257	0.727	0.331
1:22:32	1.115	0.863	0.695	0.315	0.502	0.261	0.732	0.335
1:23:32	1.113	0.871	0.707	0.321	0.509	0.266	0.736	0.341

1:24:32	1.107	0.878	0.706	0.326	0.512	0.272	0.745	0.346
1:25:32	1.107	0.88	0.719	0.329	0.519	0.277	0.746	0.352
1:26:32	1.112	0.885	0.719	0.338	0.522	0.279	0.751	0.354
1:27:32	1.113	0.882	0.724	0.338	0.528	0.285	0.76	0.357
1:28:32	1.117	0.883	0.732	0.344	0.532	0.291	0.765	0.362
1:29:32	1.12	0.889	0.729	0.348	0.538	0.297	0.771	0.369
1:30:32	1.12	0.902	0.738	0.354	0.54	0.303	0.772	0.375
1:31:32	1.124	0.907	0.738	0.357	0.546	0.31	0.776	0.377
1:32:32	1.119	0.908	0.742	0.362	0.551	0.316	0.783	0.383
1:33:32	1.127	0.91	0.748	0.37	0.557	0.319	0.786	0.388
1:34:32	1.122	0.916	0.764	0.371	0.561	0.325	0.791	0.397
1:35:32	1.13	0.909	0.768	0.376	0.567	0.326	0.798	0.405
1:36:32	1.129	0.914	0.759	0.378	0.568	0.332	0.8	0.405
1:37:32	1.127	0.917	0.775	0.387	0.576	0.339	0.806	0.413
1:38:32	1.128	0.917	0.775	0.387	0.581	0.343	0.812	0.417
1:39:32	1.127	0.923	0.784	0.396	0.585	0.347	0.813	0.422
1:40:32	1.125	0.924	0.777	0.4	0.585	0.35	0.819	0.424

Kinetic read	E359A	E359A+rutin	E359K	E359K+rutin	Rutin
0:00:32	0.043	0.046	0.04	0.045	0.034
0:01:32	0.045	0.046	0.041	0.045	0.034
0:02:32	0.044	0.052	0.041	0.045	0.034
0:03:32	0.045	0.047	0.041	0.044	0.034

0:04:32	0.046	0.048	0.041	0.044	0.034
0:05:32	0.046	0.046	0.041	0.044	0.034
0:06:32	0.046	0.046	0.041	0.044	0.034
0:07:32	0.046	0.046	0.041	0.044	0.034
0:08:32	0.046	0.046	0.041	0.044	0.034
0:09:32	0.045	0.046	0.043	0.043	0.034
0:10:32	0.045	0.046	0.047	0.043	0.034
0:11:32	0.045	0.046	0.051	0.043	0.034
0:12:32	0.045	0.046	0.058	0.043	0.034
0:13:32	0.046	0.046	0.066	0.043	0.034
0:14:32	0.05	0.045	0.074	0.043	0.034
0:15:32	0.055	0.046	0.084	0.044	0.034
0:16:32	0.062	0.045	0.093	0.043	0.034
0:17:32	0.069	0.045	0.103	0.043	0.034
0:18:32	0.078	0.048	0.114	0.043	0.034
0:19:32	0.086	0.045	0.124	0.043	0.034
0:20:32	0.096	0.046	0.135	0.043	0.034
0:21:32	0.106	0.046	0.145	0.043	0.034
0:22:32	0.117	0.046	0.157	0.043	0.034
0:23:32	0.128	0.045	0.167	0.043	0.034
0:24:32	0.137	0.045	0.178	0.043	0.034
0:25:32	0.149	0.046	0.192	0.044	0.034
0:26:32	0.162	0.046	0.2	0.043	0.034
0:27:32	0.171	0.045	0.21	0.044	0.034
0:28:32	0.182	0.046	0.221	0.044	0.034
0:29:32	0.192	0.046	0.231	0.044	0.034
0:30:32	0.204	0.046	0.242	0.045	0.034
0:31:32	0.216	0.046	0.253	0.047	0.034
0:32:32	0.226	0.047	0.264	0.05	0.034
0:33:32	0.237	0.048	0.272	0.053	0.034
0:34:32	0.248	0.051	0.284	0.056	0.034
0:35:32	0.258	0.054	0.294	0.06	0.034
0:36:32	0.269	0.058	0.302	0.064	0.034
0:37:32	0.279	0.061	0.311	0.068	0.034
0:38:32	0.29	0.066	0.321	0.072	0.034
0:39:32	0.3	0.07	0.331	0.077	0.034
0:40:32	0.309	0.075	0.341	0.082	0.034
0:41:32	0.318	0.08	0.349	0.087	0.034
0:42:32	0.329	0.085	0.36	0.092	0.034
0:43:32	0.34	0.09	0.368	0.097	0.034
0:44:32	0.35	0.096	0.376	0.103	0.034

0:45:32	0.362	0.102	0.388	0.108	0.033
0:46:32	0.368	0.107	0.396	0.114	0.034
0:47:32	0.379	0.113	0.405	0.12	0.034
0:48:32	0.385	0.119	0.415	0.125	0.034
0:49:32	0.399	0.125	0.423	0.131	0.034
0:50:32	0.404	0.131	0.43	0.137	0.034
0:51:32	0.415	0.137	0.436	0.143	0.034
0:52:32	0.424	0.144	0.446	0.149	0.034
0:53:32	0.431	0.15	0.453	0.155	0.034
0:54:32	0.437	0.157	0.46	0.162	0.034
0:55:32	0.448	0.164	0.472	0.167	0.034
0:56:32	0.453	0.169	0.477	0.172	0.034
0:57:32	0.46	0.175	0.488	0.177	0.034
0:58:32	0.469	0.181	0.494	0.184	0.034
0:59:32	0.478	0.188	0.493	0.19	0.034
1:00:32	0.484	0.195	0.505	0.197	0.034
1:01:32	0.489	0.201	0.516	0.205	0.034
1:02:32	0.493	0.207	0.522	0.21	0.034
1:03:32	0.5	0.214	0.529	0.215	0.034
1:04:32	0.511	0.22	0.533	0.223	0.034
1:05:32	0.518	0.226	0.539	0.228	0.034
1:06:32	0.525	0.232	0.543	0.234	0.034
1:07:32	0.533	0.239	0.54	0.239	0.034
1:08:32	0.537	0.245	0.557	0.246	0.034
1:09:32	0.542	0.25	0.552	0.249	0.034
1:10:32	0.549	0.257	0.558	0.257	0.034
1:11:32	0.557	0.262	0.568	0.261	0.034
1:12:32	0.564	0.268	0.573	0.268	0.034
1:13:32	0.571	0.275	0.581	0.273	0.034
1:14:32	0.577	0.28	0.587	0.276	0.034
1:15:32	0.585	0.285	0.587	0.28	0.034
1:16:32	0.585	0.292	0.594	0.288	0.034
1:17:32	0.595	0.298	0.6	0.297	0.034
1:18:32	0.598	0.303	0.604	0.302	0.034
1:19:32	0.604	0.311	0.604	0.308	0.034
1:20:32	0.615	0.318	0.612	0.313	0.034
1:21:32	0.617	0.322	0.618	0.317	0.034
1:22:32	0.626	0.33	0.624	0.323	0.034
1:23:32	0.63	0.334	0.627	0.324	0.034
1:24:32	0.634	0.339	0.633	0.331	0.034
1:25:32	0.639	0.343	0.642	0.336	0.034

1:26:32	0.648	0.349	0.648	0.338	0.034
1:27:32	0.654	0.354	0.646	0.343	0.034
1:28:32	0.661	0.358	0.651	0.35	0.034
1:29:32	0.663	0.364	0.658	0.356	0.034
1:30:32	0.671	0.366	0.66	0.358	0.034
1:31:32	0.678	0.374	0.664	0.365	0.034
1:32:32	0.675	0.378	0.667	0.367	0.034
1:33:32	0.686	0.382	0.668	0.376	0.034
1:34:32	0.691	0.387	0.669	0.38	0.034
1:35:32	0.691	0.392	0.679	0.381	0.034
1:36:32	0.695	0.395	0.682	0.385	0.034
1:37:32	0.703	0.401	0.686	0.389	0.034
1:38:32	0.705	0.405	0.691	0.396	0.034
1:39:32	0.705	0.407	0.696	0.399	0.034
1:40:32	0.711	0.416	0.698	0.4	0.034

3. Raw data of Fig. 6
1) Fig. 6A

**Kinetic read**	**PDI**	**PDI+rutin**	**PDI+kaempfertrin**	**PDI+tiliroside**	**PDI+2'-O-galloylhyperin**
0:00:32	0.048	0.073	0.048	0.044	0.057
0:01:32	0.056	0.073	0.049	0.045	0.057
0:02:32	0.085	0.073	0.051	0.047	0.058
0:03:32	0.131	0.073	0.054	0.047	0.058
0:04:32	0.181	0.074	0.063	0.047	0.058
0:05:32	0.233	0.073	0.079	0.047	0.058
0:06:32	0.283	0.073	0.099	0.047	0.057
0:07:32	0.331	0.073	0.122	0.047	0.057
0:08:32	0.379	0.073	0.146	0.048	0.058
0:09:32	0.42	0.079	0.173	0.052	0.058

	1				
0:10:32	0.466	0.087	0.198	0.059	0.058
0:11:32	0.503	0.101	0.224	0.068	0.059
0:12:32	0.546	0.116	0.251	0.08	0.063
0:13:32	0.578	0.134	0.275	0.094	0.068
0:14:32	0.618	0.15	0.306	0.11	0.074
0:15:32	0.652	0.168	0.33	0.128	0.082
0:16:32	0.682	0.185	0.354	0.144	0.09
0:17:32	0.708	0.202	0.377	0.161	0.101
0:18:32	0.736	0.221	0.406	0.178	0.11
0:19:32	0.76	0.238	0.427	0.198	0.122
0:20:32	0.78	0.255	0.45	0.214	0.135
0:21:32	0.805	0.276	0.473	0.23	0.146
0:22:32	0.814	0.29	0.499	0.25	0.157
0:23:32	0.836	0.31	0.521	0.271	0.173
0:24:32	0.859	0.325	0.539	0.289	0.186
0:25:32	0.875	0.342	0.557	0.304	0.2
0:26:32	0.889	0.361	0.571	0.323	0.212
0:27:32	0.907	0.377	0.58	0.338	0.225
0:28:32	0.913	0.394	0.598	0.353	0.242
0:29:32	0.932	0.407	0.628	0.363	0.25
0:30:32	0.946	0.424	0.651	0.382	0.255

0:31:32	0.961	0.438	0.672	0.397	0.265
0:32:32	0.975	0.45	0.691	0.412	0.282
0:33:32	0.981	0.468	0.71	0.426	0.294
0:34:32	0.99	0.478	0.726	0.441	0.307
0:35:32	1.008	0.491	0.738	0.457	0.314
0:36:32	1.016	0.508	0.75	0.467	0.326
0:37:32	1.026	0.518	0.759	0.486	0.336
0:38:32	1.032	0.534	0.767	0.496	0.348
0:39:32	1.038	0.545	0.782	0.512	0.358
0:40:32	1.044	0.556	0.8	0.52	0.37
0:41:32	1.047	0.569	0.814	0.534	0.382
0:42:32	1.048	0.58	0.828	0.547	0.399
0:43:32	1.053	0.586	0.833	0.558	0.414
0:44:32	1.064	0.597	0.843	0.569	0.42
0:45:32	1.063	0.603	0.856	0.578	0.435
0:46:32	1.073	0.605	0.874	0.593	0.442
0:47:32	1.072	0.61	0.875	0.6	0.452
0:48:32	1.077	0.619	0.882	0.616	0.46
0:49:32	1.075	0.633	0.896	0.621	0.471
0:50:32	1.082	0.659	0.905	0.64	0.478
0:51:32	1.08	0.662	0.906	0.641	0.488
0:52:32	1.08	0.671	0.919	0.647	0.493

	6				
0:53:32	1.085	0.686	0.923	0.657	0.509
0:54:32	1.091	0.691	0.928	0.67	0.52
0:55:32	1.092	0.691	0.929	0.677	0.528
0:56:32	1.088	0.7	0.937	0.692	0.53
0:57:32	1.091	0.707	0.945	0.694	0.543
0:58:32	1.096	0.717	0.954	0.697	0.553
0:59:32	1.098	0.728	0.949	0.706	0.564
1:00:32	1.099	0.731	0.967	0.707	0.576
1:01:32	1.098	0.742	0.961	0.716	0.581
1:02:32	1.102	0.752	0.956	0.725	0.584
1:03:32	1.101	0.762	0.97	0.726	0.592
1:04:32	1.103	0.759	0.967	0.737	0.6
1:05:32	1.105	0.768	0.96	0.755	0.609
1:06:32	1.107	0.784	0.964	0.756	0.62
1:07:32	1.105	0.785	0.962	0.762	0.625
1:08:32	1.108	0.787	0.972	0.78	0.637
1:09:32	1.105	0.794	0.969	0.781	0.64
1:10:32	1.106	0.804	0.971	0.785	0.649
1:11:32	1.109	0.814	0.974	0.789	0.658
1:12:32	1.111	0.812	0.98	0.802	0.66
1:13:32	1.111	0.823	0.98	0.808	0.672

1:14:32	1.111	0.829	0.98	0.812	0.676
1:15:32	1.111	0.831	0.98	0.806	0.681
1:16:32	1.108	0.845	0.986	0.817	0.687
1:17:32	1.112	0.834	0.991	0.826	0.695
1:18:32	1.116	0.842	0.993	0.83	0.706
1:19:32	1.114	0.85	0.983	0.839	0.712
1:20:32	1.116	0.863	0.992	0.835	0.723
1:21:32	1.115	0.873	0.979	0.844	0.726
1:22:32	1.115	0.863	0.994	0.855	0.729
1:23:32	1.113	0.871	0.995	0.852	0.737
1:24:32	1.107	0.878	0.992	0.859	0.743
1:25:32	1.107	0.88	0.995	0.873	0.746
1:26:32	1.112	0.885	0.991	0.866	0.761
1:27:32	1.113	0.882	0.99	0.874	0.764
1:28:32	1.117	0.883	1.003	0.878	0.761
1:29:32	1.12	0.889	0.989	0.883	0.771
1:30:32	1.12	0.902	0.991	0.884	0.771
1:31:32	1.124	0.907	0.992	0.889	0.782
1:32:32	1.119	0.908	0.996	0.901	0.787
1:33:32	1.127	0.91	0.993	0.894	0.792
1:34:32	1.122	0.916	0.986	0.899	0.798
1:35:32	1.13	0.909	0.992	0.907	0.805
1:36:32	1.129	0.914	0.991	0.913	0.805
1:37:32	1.127	0.917	0.993	0.91	0.815
1:38:32	1.128	0.917	0.999	0.914	0.813
1:39:32	1.127	0.923	1.001	0.917	0.826
1:40:32	1.125	0.924	0.998	0.925	0.825

2) Fig. 6B

Kinetic read	MBP-PDI-b'xa' (WT)	WT+rutin	WT+kaempferitrin	WT+tiliroside	WT+2’-O-galloylhyperin
0:00:32	0.042	0.047	0.06	0.049	0.065
0:01:32	0.042	0.047	0.055	0.049	0.065
0:02:32	0.042	0.048	0.055	0.05	0.066
0:03:32	0.042	0.048	0.054	0.049	0.065
0:04:32	0.042	0.048	0.054	0.05	0.064
0:05:32	0.043	0.048	0.054	0.049	0.065
0:06:32	0.043	0.049	0.054	0.049	0.064
0:07:32	0.043	0.048	0.054	0.049	0.064
0:08:32	0.043	0.048	0.054	0.049	0.064
0:09:32	0.046	0.048	0.054	0.049	0.064
0:10:32	0.053	0.049	0.055	0.049	0.064
0:11:32	0.062	0.048	0.054	0.048	0.063
0:12:32	0.073	0.048	0.054	0.048	0.064
0:13:32	0.085	0.048	0.055	0.048	0.063
0:14:32	0.098	0.049	0.055	0.048	0.065
0:15:32	0.112	0.049	0.057	0.048	0.063
0:16:32	0.125	0.049	0.059	0.048	0.062
0:17:32	0.138	0.048	0.062	0.048	0.062
0:18:32	0.153	0.049	0.065	0.05	0.062

0:19:32	0.167	0.049	0.069	0.048	0.062
0:20:32	0.182	0.049	0.074	0.048	0.062
0:21:32	0.197	0.051	0.079	0.049	0.062
0:22:32	0.21	0.055	0.085	0.048	0.062
0:23:32	0.225	0.06	0.091	0.049	0.063
0:24:32	0.239	0.067	0.099	0.05	0.063
0:25:32	0.254	0.073	0.106	0.051	0.063
0:26:32	0.268	0.081	0.113	0.053	0.064
0:27:32	0.282	0.088	0.121	0.056	0.067
0:28:32	0.297	0.096	0.128	0.058	0.068
0:29:32	0.307	0.105	0.136	0.062	0.07
0:30:32	0.323	0.114	0.141	0.066	0.073
0:31:32	0.337	0.123	0.15	0.07	0.076
0:32:32	0.348	0.133	0.155	0.075	0.08
0:33:32	0.36	0.141	0.162	0.081	0.083
0:34:32	0.369	0.151	0.171	0.087	0.086
0:35:32	0.377	0.162	0.179	0.092	0.09
0:36:32	0.39	0.172	0.187	0.098	0.095
0:37:32	0.402	0.181	0.193	0.105	0.099
0:38:32	0.404	0.192	0.201	0.112	0.105
0:39:3	0.417	0.202	0.208	0.119	0.11

2					
0:40:32	0.426	0.212	0.218	0.127	0.117
0:41:32	0.442	0.222	0.223	0.134	0.122
0:42:32	0.442	0.232	0.235	0.141	0.128
0:43:32	0.462	0.242	0.237	0.149	0.133
0:44:32	0.472	0.252	0.246	0.157	0.138
0:45:32	0.479	0.262	0.258	0.165	0.144
0:46:32	0.491	0.272	0.263	0.172	0.151
0:47:32	0.497	0.281	0.271	0.181	0.158
0:48:32	0.516	0.288	0.278	0.189	0.165
0:49:32	0.525	0.299	0.282	0.196	0.171
0:50:32	0.535	0.307	0.297	0.204	0.177
0:51:32	0.537	0.319	0.297	0.214	0.187
0:52:32	0.546	0.324	0.31	0.221	0.191
0:53:32	0.562	0.33	0.314	0.23	0.198
0:54:32	0.57	0.336	0.32	0.238	0.204
0:55:32	0.579	0.345	0.323	0.242	0.21
0:56:32	0.58	0.355	0.333	0.25	0.219
0:57:32	0.6	0.361	0.338	0.258	0.227
0:58:32	0.602	0.371	0.345	0.268	0.233
0:59:32	0.609	0.378	0.351	0.275	0.24

1:00:32	0.619	0.387	0.357	0.281	0.248
1:01:32	0.619	0.396	0.366	0.287	0.254
1:02:32	0.629	0.403	0.372	0.298	0.263
1:03:32	0.632	0.409	0.374	0.303	0.266
1:04:32	0.645	0.413	0.382	0.308	0.27
1:05:32	0.643	0.424	0.39	0.313	0.278
1:06:32	0.654	0.43	0.395	0.323	0.285
1:07:32	0.662	0.433	0.395	0.332	0.29
1:08:32	0.666	0.44	0.409	0.335	0.296
1:09:32	0.669	0.446	0.413	0.344	0.303
1:10:32	0.683	0.457	0.418	0.348	0.307
1:11:32	0.675	0.463	0.422	0.353	0.314
1:12:32	0.691	0.466	0.431	0.358	0.322
1:13:32	0.698	0.469	0.434	0.365	0.325
1:14:32	0.7	0.475	0.438	0.377	0.331
1:15:32	0.71	0.48	0.445	0.381	0.34
1:16:32	0.715	0.484	0.448	0.389	0.344
1:17:32	0.72	0.495	0.455	0.39	0.351
1:18:32	0.722	0.495	0.461	0.4	0.355
1:19:32	0.727	0.502	0.466	0.403	0.359
1:20:3	0.746	0.502	0.472	0.406	0.364

2					
1:21:32	0.734	0.519	0.476	0.412	0.369
1:22:32	0.741	0.513	0.481	0.416	0.375
1:23:32	0.739	0.516	0.486	0.423	0.382
1:24:32	0.756	0.528	0.489	0.43	0.386
1:25:32	0.759	0.536	0.495	0.432	0.389
1:26:32	0.758	0.538	0.501	0.441	0.394
1:27:32	0.759	0.538	0.502	0.447	0.401
1:28:32	0.783	0.544	0.511	0.449	0.405
1:29:32	0.781	0.548	0.514	0.454	0.41
1:30:32	0.779	0.549	0.516	0.457	0.413
1:31:32	0.796	0.553	0.52	0.462	0.414
1:32:32	0.787	0.554	0.522	0.466	0.424
1:33:32	0.804	0.558	0.523	0.471	0.428
1:34:32	0.805	0.564	0.533	0.475	0.434
1:35:32	0.803	0.574	0.537	0.482	0.434
1:36:32	0.801	0.57	0.55	0.482	0.443
1:37:32	0.811	0.577	0.546	0.484	0.445
1:38:32	0.808	0.579	0.55	0.493	0.453
1:39:32	0.831	0.58	0.555	0.499	0.453
1:40:32	0.829	0.585	0.56	0.5	0.462

4. Raw data of Fig. S3

1) PDI

Concentration (μM)	2.27E-07	6.8E-07	2.04E-06	6.11E-06	0.0000183	0.000055	0.000165	0.004455
Fluorescence	223	290.3	272	449	443.3	1370	1737.3	1812
Fluorescence	238	259.3	263	278	1164.3	1432	1267.3	1800
Fluorescence	175	183.3	236	397	777.3	663	772.3	1471

2) MBP-PDI-b’x

Concentration (μM)	2.27E-07	6.8E-07	2.04E-06	6.11E-06	0.0000183	0.000055	0.000165	0.000495	0.004455
Fluorescence	266	186.3	215	356	872.3	2501	3723.3	2613	2251
Fluorescence	171	100.3	256	397	991.3	2228	2463.3	2857	2500
Fluorescence	203	163.3	179	628	1110.3	3385	2718.3	2389	2342

3) MBP-PDI-b’xa’

Concentration (μM)	2.27E-07	6.8E-07	2.04E-06	6.11E-06	0.0000183	0.000055	0.000165	0.000495	0.004455
Fluorescence	107	212.3	250	444	770.3	1073	1140.3	1373	1563
Fluorescence	249	214.3	200	135	911.3	1060	756.3	1277	1100
Fluorescence	149	181.3	244	388	322.3	774	1238.3	1331	1415

4) MBP-mPDI-b’x

Concentration (μM)	2.27E-07	6.8E-07	2.04E-06	6.11E-06	0.0000183	0.000055	0.001485	0.004455
Fluorescence	110	236.3	251	407	1137.3	1850	1718.7	2011
Fluorescence	233	147.3	186	589	953.3	1071	1883.7	1949
Fluorescence	224	207.3	261	534	1030.3	2610	1720.7	2214

5) MBP-mPDI-b’xa’

Concentration (μM)	2.27E-07	6.8E-07	2.04E-06	6.11E-06	0.0000183	0.000055	0.000165	0.000495	0.004455
Fluorescence	101	227.3	140	425	938.3	1005	1183.3	1760	1672
Fluorescence	223	224.3	235	429	864.3	633	1573.3	1383	1527
Fluorescence	277	369.3	265	501	758.3	1332	1424.3	916	1670

5. Raw data of Fig. S4

**Wavelength**	**rutin**	**MBP-PNGase+rutin**	**MBP-SpoIVB+rutin**	**MBP-MOC1+rutin**	**MBP-PNGase**	**MBP-SpoIVB**	**MBP-MOC**	**Buffer**
460	132	145	155	99	147	131	111	109
470	155	144	150	111	147	120	106	93
480	151	188	180	139	141	149	115	122
490	227	219	230	187	169	185	175	132
500	269	234	276	227	227	211	182	168
510	295	290	319	247	193	209	169	144
520	312	296	262	227	169	171	143	112
530	320	282	262	235	132	128	111	104
540	309	299	288	285	109	134	104	78
550	300	270	269	238	94	89	93	71
560	290	268	264	223	99	90	78	54
570	254	240	229	197	81	87	70	43

580	248	225	207	181	71	72	53	49
590	203	184	178	163	50	65	55	35
600	165	173	169	129	34	57	38	26
610	143	153	127	128	44	35	28	37
620	109	123	104	110	41	21	25	33
630	100	99	98	79	19	23	9	10
640	107	100	91	75	19	13	10	13
650	93	68	88	66	28	18	10	8
660	102	91	105	62	7	11	13	15
670	70	59	87	59	30	7	10	11
680	45	59	56	31	15	9	7	7
690	45	23	60	28	4	8	1	0
700	25	54	67	35	7	7	4	0

6. Raw data of Fig. S7

**Kinetic read**	**H354A+rutin**	**H354A+rutin**	**H354A+rutin**
0:00:32	0.035	0.036	0.039
0:01:32	0.035	0.035	0.045
0:02:32	0.035	0.035	0.041
0:03:32	0.035	0.035	0.041
0:04:32	0.035	0.036	0.041
0:05:32	0.036	0.036	0.042
0:06:32	0.036	0.036	0.043
0:07:32	0.038	0.036	0.041
0:08:32	0.036	0.036	0.042
0:09:32	0.036	0.036	0.062
0:10:32	0.036	0.037	0.053
0:11:32	0.036	0.036	0.06
0:12:32	0.036	0.036	0.041
0:13:32	0.039	0.036	0.041
0:14:32	0.036	0.037	0.042
0:15:32	0.036	0.037	0.041

0:16:32	0.039	0.038	0.048
0:17:32	0.036	0.037	0.04
0:18:32	0.037	0.037	0.04
0:19:32	0.036	0.037	0.042
0:20:32	0.037	0.038	0.041
0:21:32	0.037	0.037	0.04
0:22:32	0.036	0.037	0.06
0:23:32	0.036	0.037	0.04
0:24:32	0.036	0.037	0.04
0:25:32	0.036	0.037	0.04
0:26:32	0.039	0.037	0.041
0:27:32	0.04	0.037	0.04
0:28:32	0.038	0.038	0.04
0:29:32	0.039	0.037	0.04
0:30:32	0.037	0.038	0.041
0:31:32	0.039	0.038	0.04
0:32:32	0.041	0.039	0.046
0:33:32	0.043	0.04	0.043
0:34:32	0.047	0.042	0.044
0:35:32	0.05	0.045	0.046
0:36:32	0.053	0.048	0.05
0:37:32	0.057	0.052	0.053
0:38:32	0.062	0.056	0.057
0:39:32	0.065	0.06	0.06
0:40:32	0.07	0.064	0.065
0:41:32	0.075	0.068	0.074
0:42:32	0.08	0.072	0.085
0:43:32	0.084	0.077	0.081
0:44:32	0.089	0.082	0.083
0:45:32	0.095	0.088	0.087
0:46:32	0.1	0.092	0.104
0:47:32	0.108	0.098	0.099
0:48:32	0.111	0.104	0.103
0:49:32	0.118	0.109	0.108
0:50:32	0.122	0.114	0.114
0:51:32	0.128	0.12	0.119
0:52:32	0.134	0.126	0.13
0:53:32	0.139	0.132	0.13
0:54:32	0.146	0.137	0.136
0:55:32	0.154	0.143	0.142
0:56:32	0.158	0.15	0.147

0:57:32	0.165	0.154	0.163
0:58:32	0.169	0.16	0.159
0:59:32	0.174	0.167	0.164
1:00:32	0.18	0.172	0.175
1:01:32	0.191	0.177	0.176
1:02:32	0.193	0.183	0.181
1:03:32	0.199	0.189	0.188
1:04:32	0.205	0.195	0.193
1:05:32	0.21	0.201	0.198
1:06:32	0.215	0.206	0.203
1:07:32	0.221	0.212	0.208
1:08:32	0.227	0.217	0.214
1:09:32	0.237	0.222	0.222
1:10:32	0.238	0.227	0.229
1:11:32	0.244	0.234	0.234
1:12:32	0.249	0.239	0.234
1:13:32	0.255	0.245	0.241
1:14:32	0.26	0.252	0.247
1:15:32	0.265	0.255	0.251
1:16:32	0.274	0.261	0.257
1:17:32	0.276	0.267	0.265
1:18:32	0.281	0.271	0.27
1:19:32	0.288	0.276	0.273
1:20:32	0.291	0.282	0.274
1:21:32	0.296	0.286	0.281
1:22:32	0.305	0.293	0.291
1:23:32	0.309	0.297	0.294
1:24:32	0.311	0.304	0.296
1:25:32	0.316	0.307	0.304
1:26:32	0.322	0.311	0.314
1:27:32	0.326	0.318	0.312
1:28:32	0.333	0.323	0.319
1:29:32	0.337	0.327	0.323
1:30:32	0.343	0.331	0.328
1:31:32	0.346	0.334	0.331
1:32:32	0.352	0.341	0.337
1:33:32	0.357	0.344	0.341
1:34:32	0.363	0.348	0.345
1:35:32	0.365	0.353	0.353
1:36:32	0.368	0.358	0.356
1:37:32	0.373	0.362	0.358

1:38:32	0.378	0.367	0.358
1:39:32	0.383	0.369	0.363
1:40:32	0.385	0.376	0.368

7. Raw data of Fig. S8

**Kinetic read**	**Rutin+insulin**	**Kaempferitrin+insulin**	**Tiliroside+insulin**	**2'-O-galloylhyperin+insulin**
0:00:32	0.034	0.039	0.043	0.045
0:01:32	0.034	0.039	0.043	0.045
0:02:32	0.034	0.039	0.043	0.045
0:03:32	0.034	0.039	0.043	0.045
0:04:32	0.034	0.039	0.043	0.045
0:05:32	0.034	0.039	0.043	0.045
0:06:32	0.034	0.039	0.043	0.045
0:07:32	0.034	0.039	0.043	0.045
0:08:32	0.034	0.039	0.043	0.045
0:09:32	0.034	0.039	0.043	0.045
0:10:32	0.034	0.039	0.043	0.045
0:11:32	0.034	0.039	0.043	0.045
0:12:32	0.034	0.039	0.043	0.045
0:13:32	0.034	0.039	0.043	0.045
0:14:32	0.034	0.039	0.043	0.045
0:15:32	0.034	0.039	0.043	0.045

0:16:32	0.034	0.039	0.043	0.045
0:17:32	0.034	0.039	0.043	0.045
0:18:32	0.034	0.039	0.043	0.045
0:19:32	0.034	0.039	0.043	0.045
0:20:32	0.034	0.039	0.043	0.045
0:21:32	0.034	0.039	0.043	0.045
0:22:32	0.034	0.039	0.043	0.045
0:23:32	0.034	0.039	0.043	0.045
0:24:32	0.034	0.039	0.043	0.044
0:25:32	0.034	0.039	0.043	0.045
0:26:32	0.034	0.039	0.043	0.045
0:27:32	0.034	0.039	0.043	0.045
0:28:32	0.034	0.039	0.043	0.045
0:29:32	0.034	0.039	0.043	0.045
0:30:32	0.034	0.039	0.043	0.045
0:31:32	0.034	0.039	0.043	0.045
0:32:32	0.034	0.039	0.043	0.045
0:33:32	0.034	0.039	0.043	0.045
0:34:32	0.034	0.039	0.043	0.045
0:35:32	0.034	0.039	0.043	0.045
0:36:3	0.034	0.039	0.043	0.045

2				
0:37:32	0.034	0.039	0.043	0.045
0:38:32	0.034	0.039	0.043	0.044
0:39:32	0.034	0.039	0.043	0.045
0:40:32	0.034	0.039	0.043	0.045
0:41:32	0.034	0.039	0.043	0.045
0:42:32	0.034	0.039	0.043	0.045
0:43:32	0.034	0.039	0.043	0.045
0:44:32	0.034	0.039	0.043	0.045
0:45:32	0.033	0.039	0.043	0.045
0:46:32	0.034	0.039	0.043	0.045
0:47:32	0.034	0.039	0.043	0.045
0:48:32	0.034	0.039	0.043	0.045
0:49:32	0.034	0.039	0.043	0.045
0:50:32	0.034	0.039	0.043	0.044
0:51:32	0.034	0.039	0.043	0.044
0:52:32	0.034	0.039	0.043	0.045
0:53:32	0.034	0.039	0.043	0.045
0:54:32	0.034	0.039	0.043	0.045
0:55:32	0.034	0.039	0.043	0.045
0:56:32	0.034	0.039	0.043	0.045

0:57:32	0.034	0.039	0.043	0.045
0:58:32	0.034	0.039	0.043	0.045
0:59:32	0.034	0.039	0.043	0.045
1:00:32	0.034	0.039	0.043	0.045
1:01:32	0.034	0.039	0.043	0.045
1:02:32	0.034	0.039	0.043	0.045
1:03:32	0.034	0.039	0.043	0.045
1:04:32	0.034	0.039	0.043	0.045
1:05:32	0.034	0.039	0.043	0.045
1:06:32	0.034	0.039	0.043	0.045
1:07:32	0.034	0.039	0.043	0.045
1:08:32	0.034	0.039	0.043	0.045
1:09:32	0.034	0.039	0.043	0.044
1:10:32	0.034	0.039	0.043	0.045
1:11:32	0.034	0.039	0.043	0.045
1:12:32	0.034	0.039	0.043	0.045
1:13:32	0.034	0.04	0.043	0.045
1:14:32	0.034	0.039	0.043	0.045
1:15:32	0.034	0.039	0.043	0.045
1:16:32	0.034	0.039	0.043	0.045
1:17:3	0.034	0.039	0.043	0.045

2				
1:18:32	0.034	0.039	0.043	0.045
1:19:32	0.034	0.039	0.043	0.045
1:20:32	0.034	0.039	0.043	0.045
1:21:32	0.034	0.039	0.043	0.045
1:22:32	0.034	0.039	0.043	0.045
1:23:32	0.034	0.039	0.043	0.045
1:24:32	0.034	0.039	0.043	0.045
1:25:32	0.034	0.039	0.044	0.045
1:26:32	0.034	0.039	0.043	0.045
1:27:32	0.034	0.039	0.043	0.045
1:28:32	0.034	0.039	0.043	0.045
1:29:32	0.034	0.039	0.043	0.045
1:30:32	0.034	0.04	0.044	0.045
1:31:32	0.034	0.04	0.043	0.045
1:32:32	0.034	0.04	0.043	0.045
1:33:32	0.034	0.039	0.043	0.045
1:34:32	0.034	0.04	0.043	0.045
1:35:32	0.034	0.039	0.043	0.045
1:36:32	0.034	0.04	0.043	0.045
1:37:32	0.034	0.04	0.043	0.045

1:38:32	0.034	0.039	0.043	0.045
1:39:32	0.034	0.04	0.043	0.045
1:40:32	0.034	0.04	0.043	0.045

8. Raw data of Fig. S9

**Wavelength**	**Rutin**	**WT+GSSG+Rutin**	**H354A+GSSG+Rutin**	**WT+DTT+Rutin**	**H354A+DTT+Rutin**
460	91	522	463	226	220
470	136	645	643	323	366
480	176	840	868	474	461
490	253	1110	1077	710	717
500	351	1483	1314	983	926
510	417	1702	1409	1194	1064
520	492	1714	1571	1288	1137
530	545	1683	1575	1350	1173
540	570	1546	1539	1325	1184
550	562	1419	1320	1213	1098
560	558	1288	1242	1093	1041
570	493	1137	1021	947	924
580	457	948	943	824	841
590	382	753	770	679	653
600	351	701	676	570	584
610	330	522	539	489	434
620	267	501	484	402	391
630	218	330	309	347	324
640	191	352	336	294	293
650	167	305	242	250	268
660	160	228	248	207	190
670	154	189	104	196	170
680	148	222	176	187	152
690	142	58	92	120	95
700	136	228	68	153	96
